# A systematic review of the diagnostic accuracy of artificial intelligence-based computer programs to analyze chest x-rays for pulmonary tuberculosis

**DOI:** 10.1371/journal.pone.0221339

**Published:** 2019-09-03

**Authors:** Miriam Harris, Amy Qi, Luke Jeagal, Nazi Torabi, Dick Menzies, Alexei Korobitsyn, Madhukar Pai, Ruvandhi R. Nathavitharana, Faiz Ahmad Khan

**Affiliations:** 1 Department of Epidemiology and Biostatistics, McGill University, Montreal, Canada; 2 Department of Medicine, McGill University Health Centre, Montreal, Canada; 3 Department of Medicine, Boston University–Boston Medical Center, Boston, Massachusetts, United States of America; 4 Respiratory Epidemiology and Clinical Research Unit, Montreal Chest Institute & Research Institute of the McGill University Health Centre, Montreal, Canada; 5 St. Michael's Hospital, Li Ka Shing International Healthcare Education Centre, Toronto, Canada; 6 McGill International TB Centre, Montreal, Canada; 7 Laboratories, Diagnostics & Drug Resistance Global TB Programme WHO, Geneva, Switzerland; 8 Division of Infectious Diseases, Beth Israel Deaconess Medical Center, Boston, Massachusetts, United States of America; Medical University of Vienna, AUSTRIA

## Abstract

We undertook a systematic review of the diagnostic accuracy of artificial intelligence-based software for identification of radiologic abnormalities (*computer-aided detection*, or CAD) compatible with pulmonary tuberculosis on chest x-rays (CXRs). We searched four databases for articles published between January 2005-February 2019. We summarized data on CAD type, study design, and diagnostic accuracy. We assessed risk of bias with QUADAS-2. We included 53 of the 4712 articles reviewed: 40 focused on CAD design methods (“Development” studies) and 13 focused on evaluation of CAD (“Clinical” studies). Meta-analyses were not performed due to methodological differences. Development studies were more likely to use CXR databases with greater potential for bias as compared to Clinical studies. Areas under the receiver operating characteristic curve (median AUC [IQR]) were significantly higher: in Development studies AUC: 0.88 [0.82–0.90]) versus Clinical studies (0.75 [0.66–0.87]; p-value 0.004); and with deep-learning (0.91 [0.88–0.99]) versus machine-learning (0.82 [0.75–0.89]; *p* = 0.001). We conclude that CAD programs are promising, but the majority of work thus far has been on development rather than clinical evaluation. We provide concrete suggestions on what study design elements should be improved.

## Introduction

The need to improve tuberculosis (TB) diagnostic and screening services in high-burden countries is clear: in 2016, active TB was the leading cause of death due to an infectious agent, and only 69% of the 10.4 million people that developed this disease were detected by or notified to national TB programmes [1]. In developed countries, chest x-rays (CXRs) have been used for the evaluation of persons presenting with symptoms of possible active pulmonary TB (PTB), and for screening of individuals in high risk groups, for several decades [2]. However, uptake of CXR in high TB burden countries, particularly in resource-constrained settings, has been limited [3, 4].

In recent years, there has been increasing interest in expanding access to chest radiography in order to improve TB case detection in high-burden areas [5]. However, one of the challenges is the paucity of professionals to interpret radiographic images in resource-constrained settings [6]. In recent years, advances in artificial intelligence (AI) technology and methods have led to major progress in automated image recognition by computers. AI has been applied to the analysis of radiologic images to identify abnormalities—referred to as computer-aided detection, or CAD—and represents one potential solution to overcome the personnel shortage. Two commonly used AI approaches that have been used to create CAD programs capable of reading CXRs are Machine learning (ML) and Deep Learning (DL). ML is a type of AI analysis that relies less on human specification (i.e. defining a set of variables to be included) and instead allows algorithms to decide what variables are important [7, 8]. DL is a subset of ML which attempts to model brain architecture [7]. It uses neural networks, or overlaying models, that emphasize learning increasingly meaningful representations of the data [7]. The World Health Organization (WHO) has called for greater evidence before endorsing the use of CAD in PTB diagnostic and screening pathways [5].

To date, there has been only one systematic review of CAD use for PTB detection,[9] and it was limited to reviewing the only commercially available software at the time of publication. Amongst the 5 studies included, the reviewers identified methodological limitations that prevented the pooling of results. Because the prior review was limited to studies of the single commercially available software, it excluded the vast majority of studies of CAD for detecting PTB. Hence, in order to provide a more comprehensive and expansive summary of the CAD literature we undertook an updated systematic review which included non-commercially available CAD studies. Our primary objectives were to evaluate the evidence base with regards to the estimation of the diagnostic accuracy of CAD, including assessing potential for bias, and if appropriate, to calculate pooled estimates of area under the receiver operating characteristic curves (AUC), sensitivity, and specificity. Secondary objectives were to evaluate study-level factors associated with diagnostic accuracy; including those related to the design of the study, and the type of software used (ML versus DL).

## Methods

### Design

This systematic review followed the Preferred Reporting Items for Systematic Reviews and Meta-Analyses guidelines [10]. The International Prospective Register of Systematic Reviews (PROSPERO) registration number of this protocol is CRD42018073016.

### Date source and search strategy

A search strategy was developed in consultation with an academic librarian (NT) to identify published articles in MEDLINE (Ovid), EMBASE (Ovid), PubMed, and Scopus (S1 Appendix). The search strategies included subject headings (where applicable) and text words for the concepts of pulmonary tuberculosis, computer aided diagnosis, and diagnostic accuracy. The search period was limited to papers published after January 1, 2005, and included articles published up to February 13, 2019. Studies were limited to English and French.

### Study selection

We included all published studies that used any form of computer software to analyze CXR in place of human readers, for PTB detection purposes. Studies were excluded if they reported CAD for diagnostic imaging other than CXR, or if CAD was used for diseases other than PTB. Studies reported only in conference abstracts were excluded. Four independent reviewers selected studies for inclusion (MH, AQ, LJ, FAK). Conflicts were reviewed by a third reviewer (FAK).

### Data extraction

Data were extracted using a standardized extraction form (S2 Appendix). Three reviewers performed the extraction, with one reviewer (MH) verifying all data forms completed by the second reviewer (AQ & LJ). Data collected included year of enrollment; funding sources and conflicts of interest; software name and version number; country where study was completed; CXR site and number on which the software was trained; model of CXR machine, and digitization methods; study design and patient selection methods; inclusion and exclusion criteria; microbiologic tests collected; scoring of software tools and methods of scoring selection; patient characteristics including HIV status, age, and history of TB; and diagnostic accuracy measures including sensitivity, specificity, AUC for microbiologic and radiologic references.

### Descriptive analysis

We classified studies as either Development or Clinical. Development studies primarily focused on reporting methods for creating a CAD program for PTB, and some included an assessment of diagnostic accuracy—the latter being the focus of our systematic review. Development studies were often published in engineering, computer science, medical imaging journals, or proceedings from engineering or medical imaging conferences. The development studies were further subdivided based on the type of AI technology used (ML versus DL).

Clinical studies primarily focused on the assessment of the accuracy of an already-developed CAD software. We further classified Clinical studies based on the context in which the CXR was used, using WHO terminology for categorizing usage of x-ray as either for Triage or for Screening [5]. In Triage studies, CXRs were used in a healthcare setting—hospital, or clinic—as part of the diagnostic pathway of someone with PTB symptoms. In Screening studies, CXRs were used for active case finding or prevalence surveys, where populations are screened to identify those with active TB often regardless of symptoms. The distinction was made because the prevalence of more advanced or extensive disease will be higher in the Triage setting, thereby affecting the sensitivity of CXR and hence the accuracy of CAD.

### Quality assessment with respect to the evaluation of diagnostic accuracy

The data sources used for evaluating diagnostic accuracy of CAD were databases consisting of CXRs, with each image linked to a reference standard result classifying PTB as present or absent. Some of these data sources had been used by more than one Development study. We evaluated these data sources for potential risk of bias by applying a modified Quality Assessment of Diagnostic Accuracy Studies (QUADAS)-2 approach [11]. As our interest was to assess the composition of the database itself including how PTB cases were defined, we restricted our approach to the domains of patient selection and the reference test. Because Development studies often did not provide sampling or reference details about the data sources, we sought additional information from citations that described the data sources [12–15].

We applied QUADAS-2 to all the CAD studies, assessing each study across the four domains (patient selection, the performance of the index test, performance of the reference test, and flow and timing). In all quality assessments, when the reference standard used for determining a CAD program’s diagnostic accuracy was image interpretation by a human reader instead of microbiologic testing of sputum, we judged this as a potential source of bias. This is because human interpretation of CXR is moderately specific for PTB, has variable sensitivity, is marked by limited inter-reader reliability, and the reproducibility is limited [5, 16].

### Statistical analysis

Diagnostic accuracy measures (sensitivity, specificity, AUC) were reported when available. For the studies that reported sensitivities and specificities, if two by two tables were not available, we back calculated counts based on reported accuracy measures to build forest plots. A meta-analysis was not undertaken given that different software programs were used, and for most studies the raw data necessary to meta-analyze diagnostic accuracy measures were unavailable. For studies of the most commonly reported software, CAD4TB, a meta-analysis was also not pursued due to the variability of the methods and software versions tested.

The following study-level factors were evaluated as potential determinants of the reported AUC: type of CAD study (Development vs Clinical); the method of AI software (ML versus DL); whether the same CXRs used for evaluating diagnostic accuracy were the same CXRs that had been used to train the software; the type of reference standard for PTB (microbiologically confirmed vs human interpretation of CXR image); and the degree of patient selection, index test, and reference standard bias. While the data were insufficient for a traditional meta-analysis, to identify associations between these factors and reported AUC, we compared the pooled distribution of the reported AUCs between groups defined by these study-level factors using Kruskal-Wallis tests. When studies reported more than one AUC, a mean AUC was calculated and used for this analysis. This assessment was done for the AUC but not for Sensitivity or Specificity, as the latter two were reported in too few studies to undertake a meaningful comparison of distributions.

For all Clinical studies and Development studies which reported sensitivity, specificity, and true positives, forest plots were used to visually assess heterogeneity of diagnostic accuracies.

## Results

### Study selection

**Fig 1:**
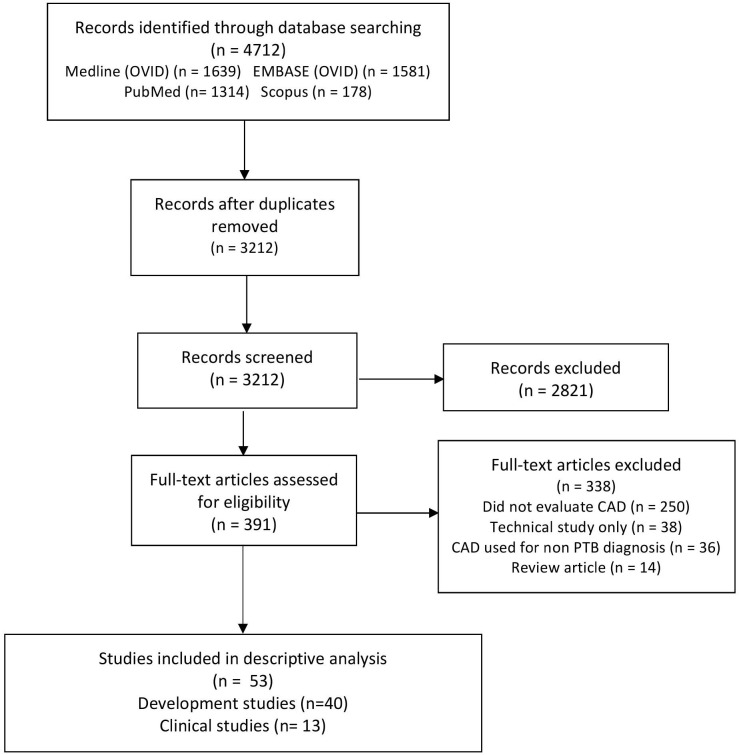
Study flow diagram. Computer aided detection (CAD).

We identified 4712 unique citations (Fig 1), of which 2821 studies were excluded at the title and abstract phase. Of the remaining 391, 338 were excluded after full-text review. Amongst the 53 included articles, 40 were classified as Development studies and 13 were classified as Clinical (Table 1). The software developers were either authors or funded the research in 9/13 (69%) of the Clinical studies [17–25], and in 100% (40/40) of the Development studies.

**Table 1:** Methods of studies included in the descriptive analysis.

Author and year	Country where CXR completed	Databases used	Computer software	Reference standard	Accuracy measures
**Development Studies**					
**Deep learning**					
Heo et al, 2019	South Korea	YU AWH	Not named	Human reader	AUC
Hwang et al, 2018	South Korea, USA, China	SNUH, BMC, KUHG, DEMC, MC, CH	DLAD	Liquid culture, NAAT, and or TB treatment	AUC,
Lakhani et al, 2017	USA, China	MC, CH, TJH, Belarus	AlexNet and GoogLeNet	Human reader	AUC, Sn, Sp
Santosh et al, 2017	USA, China, India	MC, CH, IN	Not named	Human reader	AUC, Sn, Sp
Lopes et al, 2017	USA, China	MC, CH	Not named	Human reader	AUC
Santosh et al, 2016	USA, China	MC, CH	Not named	Human reader	AUC
Hwang et al, 2016	South Korea, USA, China	KIT, MC, CH	Alexnet	Human reader	AUC
**Machine learning**					
Ilena et al, 2018	China	CH	Matlab	Human reader	Sn, Sp, TP, TN, FP, FN
Rajaraman et al, 2018	China, USA, Kenya, India	CH, MC, Kenya, IN	Not named	Human reader	AUC
Sivaramakrishnan et al, 2018	China, USA, Kenya, India	CH, MC, Kenya, IN	Custom 12-layer CNN	Human reader	AUC
Vajda et al, 2018	USA, China	MC, CH	Matlab	Human reader	AUC
Alfadhli et al, 2017	USA	MC	Not named	Human reader	AUC, Sn, TP
Fatima et al, 2017	USA	MC	Not named	Human reader	Sn, Sp
Ding et al, 2017	China, India, Kenya	Kenya, IN, CH	Not named	Human reader	NR
Hogeweg, et al, 2017	Japan, Sub-Saharan Africa	JSRT, Sub-Saharan Africa	Not named	Human reader	AUC
Udayakumar et al. 2017	USA, China	MC, CH	SVM and CBC techniques	Human reader	AUC
Maduskar et al, 2016	Zambia	Large Zambian	Not named	Human reader	AUC
Poornimadevi et al, 2016	Japan, USA	JSRT, MC	Not named	Human reader	Sn, Sp
Karargyris et al, 2016	China, Japan	JSRT, CH	Not named	Human reader	AUC
Melendez et al, 2016	Zambia	Zambian	Not named	Human reader	AUC
Melendez et al, 2015	Zambia, Tanzania, Gambia	Zambian, Tanzania, Gambian	Not named	Human reader	NR
Hogeweg et al, 2015	UK, South Africa	F&T, TB-NEAT	Not named	Human reader, Liquid culture, composite reference standard **	AUC, Sn, Sp
Giacomini et al, 2015	Brazil	Prospective, study-specific^†^	Not named	Liquid culture^+^	NR
Jaeger et al, 2015	China	CH	Not named	Human reader	NR
Requena-Mendez et al, 2015	Peru	CXR from DOT study in Peru	Not named	Human reader	NR
Jaeger et al, 2014	China, USA, Japan	JSRT, MC, CH	Not named	Human reader	AUC, Sn, Sp
Melendez et al, 2014	Zambia, South Africa	Zambian	TB-Xpredict	Human reader	AUC
Chauhan et al, 2014	India	IN	Not named	Human reader	NR
Seixas et al, 2013	Brazil	Clinical data set from another study*	Artificial Neural Network	Composite reference**	NR
Sundaram et al, 2013	Not specified	Not specified	Not named	Human reader	NR
Jaeger et al, 2012	USA, Japan	JSRT, MC	Not named	Human reader	AUC
Xu et al, 2011	Japan, Canada	JSRT, Calgary dataset	Andrews' curve	Human reader	TP, FP, FPR
Noor et al, 2011	Malaysia	Retrospective non-clinical study specific radiological	Not named	Human reader	Sn, Sp
Shen et al, 2010	Canada	JSRT, Calgary	Not named	Human reader	TP, FPR
Mouton et al, 2010	South Africa	Clinical dataset from previous study not specific to PTB	Not named	Human reader	AUC
Hogeweg et al, 2010	Sub-Saharan Africa	Sub-Saharan Africa	CAD with rib suppression	Human reader	AUC
Hogeweg et al, 2010	Not specified	Not specified	Not named	Human reader	NR
Lieberman et al, 2009	China	Prospective, study-specific^†^	Not named	Human reader	NR
Arzhaeva et al, 2009	Netherlands	F&T	Not named	Human reader	AUC
Noor et al, 2005	China, USA	MC, CH	Andrews' curve	Composite reference**	NR
**Clinical studies**					
**Machine learning**					
Koesoemadinata et al, 2018	Indonesia	Prospective study-specific^†^	CAD4TB (v 5)	Liquid culture/NAAT	AUC, Sn, Sp
Melendez et al, 2018	United Kingdom	Find & Treat	CAD4TB (v 5)	Human reader, TB treatment	AUC, Sn, Sp, TP, FP, TN, FN
Zaidi et al, 2018	Pakistan	Sehatmand Zindagi (Healthy Life)	CAD4TB (v 3.07)	NAAT	AUC, Sn, Sp
Rahman et al, 2017	Bangladesh	Prospective, study-specific^†^	CAD4TB (v 3.07)	NAAT	AUC, Sn, Sp
Melendez et al, 2017	Zambia	Zambia National TB Prevalence Survey	CAD4TB (v 5)	Human reader CXR-, Liquid culture/NAAT for CXR+	AUC, Sn, Sp
Muyoyeta et al, 2017	Zambia	Prospective, study-specific^†^	CAD4TB (v 1.08)	NAAT for CXR+, AFB Smear for CXR-	NR
Melendez et al, 2016	South Africa	TB-NEAT collaborative study	CAD4TB (v 3.07)	Liquid culture	AUC, Sn, Sp
Philipsen et al, 2015	South Africa	TB-NEAT collaborative study	CAD4TB (v 3.07)	NAAT, liquid culture	AUC, Sn, Sp
Steiner et al, 2015	Tanzania	TB REACH project	CAD4TB (v 3.07)	Human reader	AUC, Sn, Sp
Muyoyeta et al, 2015	Zambia	Prospective, study-specific^†^	CAD4TB (v 1.08)	NAAT, AFB Smear for CXR-	AUC, Sn, Sp
Breuninger et al, 2014	Tanzania	TB Cohort and TB CHILD study	CAD4TB (v 3.07)	Liquid culture, AFB smear	AUC, Sn, Sp
Muyoyeta et al, 2014	Zambia	Prospective, study-specific^†^	CAD4TB (v 1.08)	NAAT	AUC, Sn, Sp
Maduskar et al, 2013	Zambia	Prospective, study-specific^†^	CAD4TB (v 1.08)	Liquid culture, AFB smear	AUC, Sn, Sp

CXR, chest x-ray; USA, United States of America; UK, United Kingdom; AI, artificial intelligence; YU AWHE, Yonsei University annual worker's health examination; SNUH, Seoul National University Hospital; BMC, Boramae Medical Center; KUHG, Kyunghee University Hospital at Gangdong; DEMC, Daejeon Eulji Medical Center; MC, Montgomery County; CH, Shenzhen Hospital, China; IN, Indian collection New Delhi; TJH, Thomas Jefferson Hospital dataset; JSRT, Japanese Society of Radiology; KIT, Korean Institute of Tuberculosis; F&T, Find and Treat; DLAD, deep learning automatic detection; SVM, *Support vector machines;* CBC, clustering based classification; CAD, computer aided detection; NAAT, nucleic acid amplification test; AFB, acid fast bacilli; ‘+’, positive; ‘-‘, negative; AUC, area under the receiver operating curve; Sn, sensitivity; Sp, specificity; NR, not reported; TP, true positives; FP, false positives; FPR, false positive rate; TN, true negatives, FN, false negatives; ACC, accuracy

* Trajman et al. Pleural fluid ADA, IgA-ELISA and NAAT sensitivities for the diagnosis of pleural tuberculosis Study

**Composite reference: positive culture/NAAT and/or initiation of TB treatment

†In these studies the study database was developed prospectively for the specific study

### Overview of studies

Within the Development studies, 7/40 (17%) employed DL methods while the remaining 33/40 (83%) used ML approaches (Table 1) [26–65]. An important consideration when evaluating the accuracy of a CAD software, is that it should be tested using a set of CXR images that are separate from the training set (i.e. avoid testing accuracy with CXRs that were used for training, or CXRs that were not used for training but that originate from the same subset/study as those with which the program was trained). Otherwise, the evaluation is likely to overestimate the diagnostic accuracy, and will also have limited generalizability [66]. Within the Development studies that reported accuracy measures, 3/32 (12%) did not report the database used to train and test their software. Overall, the majority of studies (32/40, 80%), either used the same databases to train and test their software, or did not comment on this (Table 2). For the majority of Development studies demographic data of the study population whose CXR were used to train and evaluate CAD were not reported in detail.

**Table 2:** Accuracy measures reported by development studies.

Author and year	Database(s) used for training of CAD	Number of CXRs used for training	Database (s) used for testing CAD	Number of CXRs used for testing	Number of TB positive CXR	AUC (95% CI)	Thres-hold score	Sn (95% CI)	Sp (95% CI)
**Deep learning**									
Heo et al, 2019	YU AWHE	2000	YU AWHE	37475	1202	0.91 (NR), 0.92 (NR)^†^	NR	NR	NR
Hwang et al, 2018	SNUH	60989	SNUH, BMC, KUHG, DEMC, MC,CH	NR	6768	0.988 (0.976–0.999)	NR	0.95(SNUH), 0.94 (BMC), 1.0 (KUGH), 1.0 (DEMC), 1.0 (MC), 0.95 (CH)*	1.0 (SNUH), 0.96 (BMC), 0.91 (KUGH), 0.98 (DEMC), 0.94 (MC), 0.91 (CH)*
Lakhani et al, 2017	MC,CH, TJH, Belarus	857	MC, CH,TJ, Belarus	150	75	0.99 (0.96–1.00)	NR	0.97 (0.90–1.0)	0.95 (0.87–0.98)
Santosh et al, 2017	MC,CH, IN	976	MC,CH, IN	976	478	0.92 (MC) 0.82 (CH) 0.96 (IN)*	NR	0.88 (MC) 0.78 (CH) 0.92 (IN)*	0.81 (MC) 0.76 (CH) 0.86 (IN)*
Lopes et al, 2017	NR	NR	CHMC, CI,NR	1031	550	0.834 (CH) 0.926 (MC)*	NR	NR	NR
Santosh et al, 2016	NR	NR	CHMC, CI	878	400	0.93 (CH) & 0.88 (MC)*	NR	NR	NR
Hwang et al, 2016	KIT	9221	KIT,MC,CH	2427	NR	0.96*^+^	NR	NR	NR
**Machine learning**									
Ilena et al, 2018	CH	20	CH	30	15	NR	NR	0.67 (NR)*	0.86 (NR)*
Rajaraman et al, 2018	CH,MC, AMPATH, Kenya, IN	2073	CH,MC, Kenya,IN	2073	785	0.991 (CH) 0.962 (MC) 0.826 (Kenya) 0.965 (IN)*	NR	NR	NR
Sivaramakrishnan et al, 2018	CH,MC, Kenya, IN	1659	CH,MC, Kenya, IN	1228	785	0.926 (CH), 0.833 (MC), 0.775 (Kenya), 0.956 (IN)*	NR	NR	NR
Vajda et al, 2018	MC,CH	NR	MC,CH	814	392	0.91 (MC), 0.99 (CH)*	NR	NR	NR
Alfadhli et al, 2017	MC	97	MC	41	58	0.89*	NR	0.79*	NR
Fatima et al, 2017	MC	138	MC	138	58	NR	NR	0.83*	0.78*
Udayakumar et al.	MC,CH	NR	MC, CH	NR	NR	0.87*	NR	0.81*	0.74*
Hogeweg, et al, 2017	JSRT, Sub-Saharan Africa	NR	Sub-Saharan Africa	348	174	0.891*	NR	NR	NR
Ding et al, 2017	NR	NR	Kenya, IN,CH	NR	NR	0.949 (CH), 0.982 (IN), 0.76 (Kenya)*	NR	NR	NR
Maduskar et al, 2016	Large Zambian	629	Large Zambian	638	NR	0.9*	NR	0.83*	0.70*
Poornimadevi et al, 2016	JSRT	247	JSRT	247	NA	NR	NR	0.56*	0.36*
Karargyris et al, 2016	CH	43	JSRT,CH	NR	NR	0.93*	NR	NR	NR
Melendez et al, 2016	Zambian	461	Zambian	456	248	0.87*	0.45	NR	NR
Melendez et al, 2015	Zambian, Tanzania Gambian	1323	Zambian, Tanzania, Gambian	1313	671	0.86 (Zambia), 0.88 (Tanzania), 0.91 Gambia*	NR	NR	NR
Hogeweg et al, 2015	F&T, TB-Neat	400	F&T, TB-Neat	400	153	0.87 (0.81–0.92)(F&T), 0.74 (0.69–0.83)(TB-Neat)^#^	NR	NR	NR
Jaeger et al, 2014	MC,CH, JSRT	1000	MC,CH	753	333	0.87*	NR	0.78 (0.70–0.85)	0.81 (0.71–0.89)
Melendez et al, 2014	Zambian	461	Zambian	456	NR	0.88*	NR	NR	NR
Chauhan et al, 2014	IN	204	IN	102	153	0.96 (0.86–0.99) (DA), 0.89 (0.77–0.96) (DB)^##^	NR	0.96 (DA), 0.88 (DB)*	0. 92 DA, 0.84 (DB)*^&^
Sundaram et al, 2013	NR	95	NR	95	52	NR	NR	0.75*	0.90*
Jaeger et al, 2012	JSRT	247	MC	138	NR	0.83*	NR	NR	NR
Xu et al, 2011	JSRT, Calgary	60	JSRT, Calgary	60	NR	NR	NR	0.68*	0.68*
Noor et al, 2011	Retrospective non-clinical	90	Retrospective non-clinical	213	208	NR	NR	0.88*	0.84*
Shen et al, 2010	JSRT, Calgary	18	JSRT, Calgary	131	19	NR	NR	0.82*	NR
Mouton et al, 2010	Clinical non-TB specific	119	Clinical non-TB specific	119	NR	NR	0.78*	NR	NR
Hogeweg, et al, 2017	CRASS	348	CRASS, JSRT	498	NR	0.75*	NR	NR	NR
Arzhaeva et al, 2009	F&T	217	F&T	217*^++^	37	NR	0.83 TB-sus, 0.74 micro *^†^	NR	NR

CAD, Computer aided detection;; YU AWHE, Yonsei University annual worker's health examination; SNUH, Seoul National University Hospital; BMC, Boramae Medical Center; KUHG, Kyunghee University Hospital at Gangdong; DEMC, Daejeon Eulji Medical Center; MC, Montgomery County; CH, Shenzhen Hospital, China; IN, Indian collection New Delhi; TJH, Thomas Jefferson Hospital dataset; AMPATH, Academic Model Providing Access to Healthcare; JSRT, Japanese Society of Radiology; KIT, Korean Institute of Tuberculosis; F&T, Find and Treat; AUC, area under the receiver operating curve; 95% CI, 95 percent confidence interval; NR, not reported; DA, dataset A; DB, dataset B; Sn, sensitivity; Sp, specificity;; TP, true positives; FP, false positives; FPR, false positive rate; TB-sus, TB suspect

* No 95% CI reported

^+^Average AUC from KIT, MC, Shenzhen

^++^ 128 of the normal images were the same CXRS used in the training

# An external and radiological reference standard were used. The external reference for tuberculosis was set by an independent test not associated with the CXR; the result of a sputum culture testing for the TB-NEAT database and a combination of sputum culture testing and clinical diagnosis for the Find & Treat database

^##^ Two CXR digital image datasets, dataset A and B, were obtained from two different X-ray machines available at the National Institute of Tuberculosis and Respiratory Diseases, New Delh

^†^The database was split between TB suspect cases were re-read by a third radiologist, and if classified differently were excluded. The database contained 256 normal radiographs, 178 TB suspect radiographs, and 37 microbiologically diagnosed TB CXRs.

All Clinical studies used ML-based versions of CAD4TB. Within the triage use-case studies, 6/8 (75%) used a microbiologic reference standard on all participants [18, 19, 22, 25, 67, 68]. Within the screening studies, 4/5 (80%) used a microbiologic reference [20, 24, 69, 70]. In two Clinical studies, the CADscore was used to select which participants underwent microbiologic testing, hence the software’s diagnostic accuracy could not be assessed [17, 69]. The study populations of all the triage studies with microbiologic references were quite similar (S1 and S2 Tables). Notably, the estimated HIV and TB prevalence in the triage studies were quite high, ranging from 15% to 33%. The screening studies had lower TB prevalence compared to triage studies (S1 and S2 Tables).

### Quality assessment development studies

We first assessed the databases that were used as sources of CXR images and reference standards for the Development studies (S3 Table). Risk of selection bias was high in 2/18 (11%) of the databases. One dataset did not include PTB cases, and the other only included patients with “typical TB” images [13, 51]. Selection bias was unclear in 6/16 (38%), and low in 8/16 (50%) where consecutive enrollment either prospectively or retrospectively was used. The reference standard risk of bias was high in 10/18 (56%) studies as a human reader was used, unclear in 3/18 (17%), and low in 4/18 (22%) where a microbiologic reference was used.

**Fig 2:**
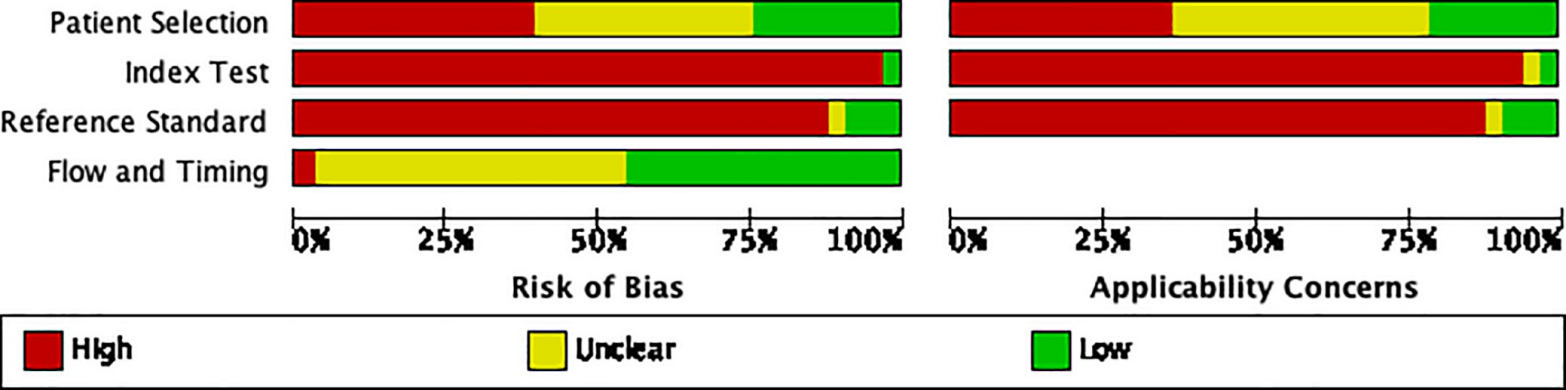
Quality assessment (QUADAS 2) graph of development studies.

The quality of the Development Studies with respect to the assessment of diagnostic accuracy is reported in Fig 2. Selection biased was largely determined by which databases were used (S3 Table). The potential for selection bias was high in 13/33 (39%) studies, unclear in 13/33 (39%), and low in 7/33 (21%). One study [62] had a pre-specified threshold score and therefore had a low risk of bias in the assessment of the index test, but the other 97% had a high risk of bias as the threshold scores were set after the analysis. Additionally, 29/33 (88%) of the studies were considered to have a high degree of bias and low degree of applicability with regards to the reference test utilized due to use of a human reader’s interpretation of CXRs. The flow and timing had low bias in 15/33 (45%) studies, in 17/33 (52%) it was unclear, and in 1/33 (3%) it was high.

### Quality assessment of clinical studies

**Fig 3:**
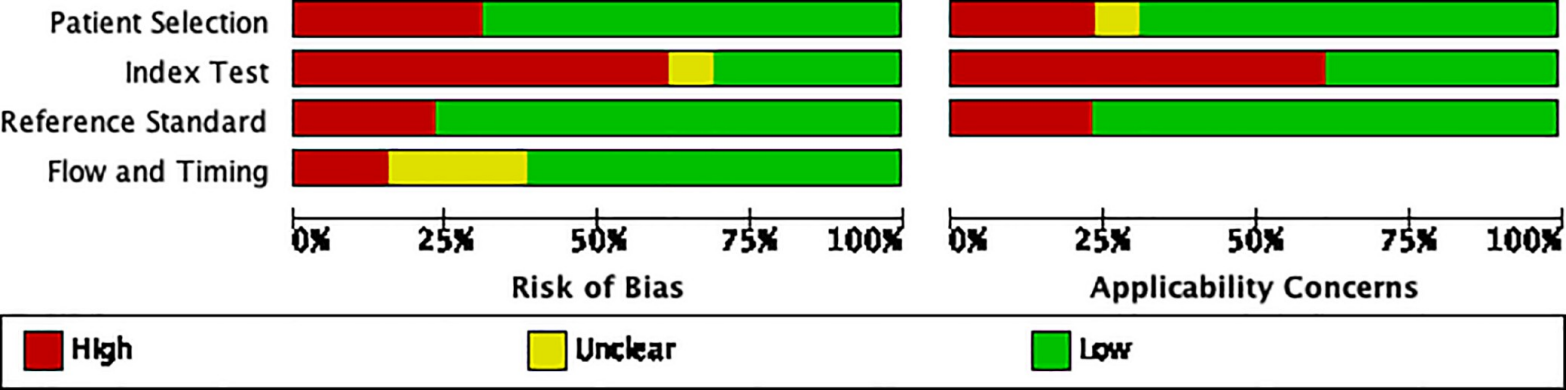
Quality assessment (QUADAS 2) graph of clinical studies.

All triage studies used a consecutive enrollment strategy, with 3/8 (38%) being prospective, 5/8 (63%) retrospective. Additional details about selection are provided in the Appendix (S2 Table). Fig 3 summarizes the QUADAS-2 assessment of the Clinical studies. There were methodological concerns that likely resulted in a high degree of selection bias in 4/13 (31%) of the studies [18, 21, 23, 68]. This was secondary to case-control design [21], and inappropriate exclusion of patients in the analysis [18, 23, 68]. The threshold score was pre-specified in only 5/13 (38%) of the studies [17, 19, 22, 25, 71]. The remainder of the studies reported threshold scores post-analysis and were therefore determined to have a high risk of bias [18, 20, 21, 23, 24, 68, 70, 72]. The majority of studies, 10/13 (77%) had low potential for bias with regards to the use and performance of the reference standard [18–20, 22–25, 70, 72]. In two studies, the CAD software was used to select patients to undergo microbiologic testing for PTB, and therefore were determined to have a high risk of bias for estimating diagnostic accuracy of CAD [17, 71]. In another study, the reference standard was human reading of the CXR which was deemed to have a high risk of bias [21]. The flow and timing had a high risk of bias in 2/10 (20%) of the studies due to CAD4TB selection of the reference standard [17, 71], was unclear in 3/10 (30%), and low in 5/10 (50%).

### Diagnostic accuracy reported in development studies

**Fig 4:**
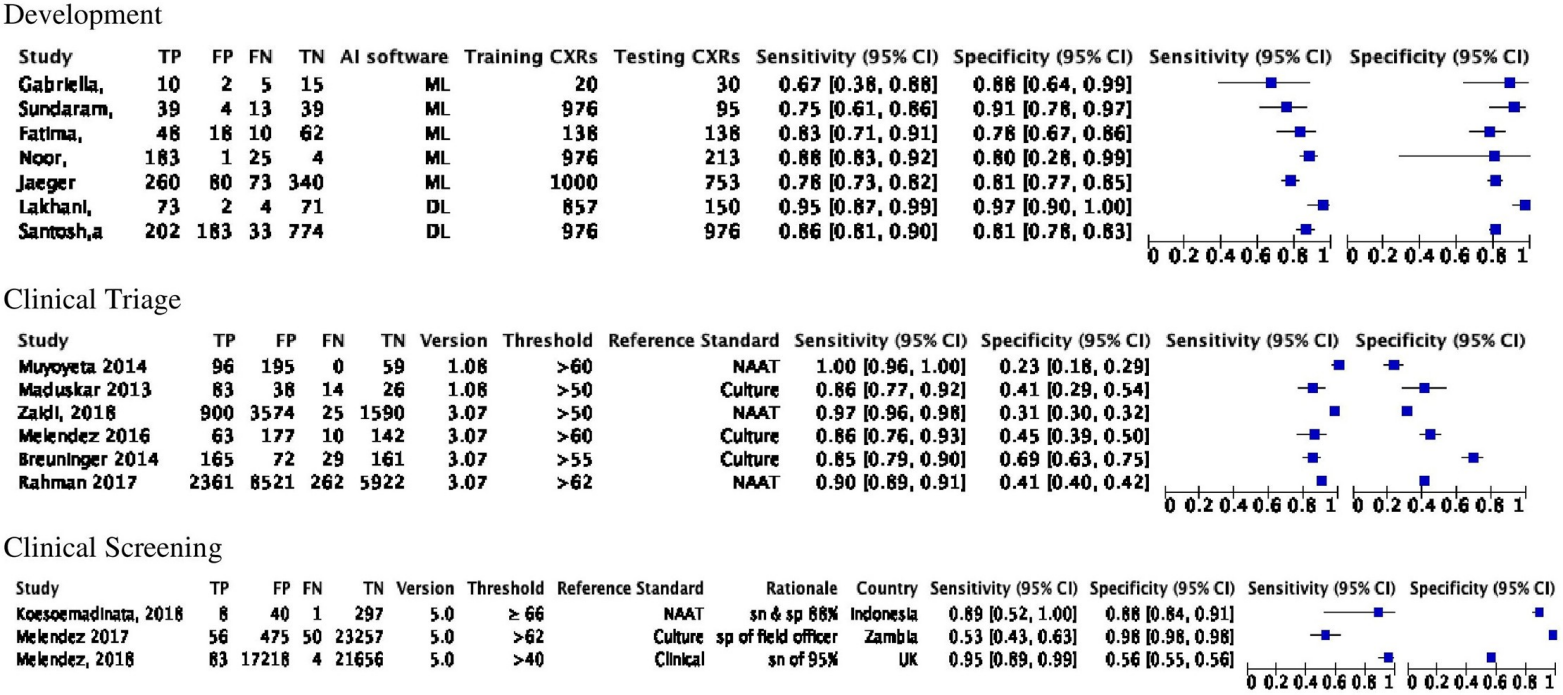
Forest plots of accuracy measures of development and CAD4TB studies. TP, true positive; FP, false positive; FN, false negative; TN, true negative; AI, artificial intelligence; CXRs, chest x-rays; ML, machine learning; DL, deep learning; CI, confidence interval; NAAT, nucleic acid amplification test.

We found 33/40 (83%) of the Development studies reported measures of accuracy for index tests. Of the 33 references that did include accuracy assessments, the AUC ranged from 0.78 to 0.99, sensitivity from 0.56 to 0.97, and specificity from 0.36 to 0.95 (Table 2). The forest plots graphically display the diagnostic heterogeneity of the sensitivity and specificity of the Development studies that published sensitivity, specificity, and the number of true positive TB cases (Fig 4).

### Diagnostic accuracy reported in clinical studies

The forest plots graphically display the diagnostic heterogeneity of the sensitivity and specificity of the triage studies that used a microbiologic reference (Fig 4). In these studies, the sensitivity ranged from 0.86 to 1.00, and specificity ranged from 0.23 to 0.69. In the screening studies, sensitivity ranged from 0.53 to 0.89 and the specificity ranged from 0.56 to 0.98. In one screening study, [21] investigators used a human reader as the reference standard and reported the sensitivity and specificity of CAD were 0.59 and 0.78, respectively. The sensitivity of CAD was higher when using NAAT as the microbiologic reference standard compared to culture. Given the methodological heterogeneity, the lack of standardized threshold scores, and the variability of software versions used, a meta-analysis was not undertaken.

### Assessment of study-level factors associated with reported AUC

**Fig 5:**
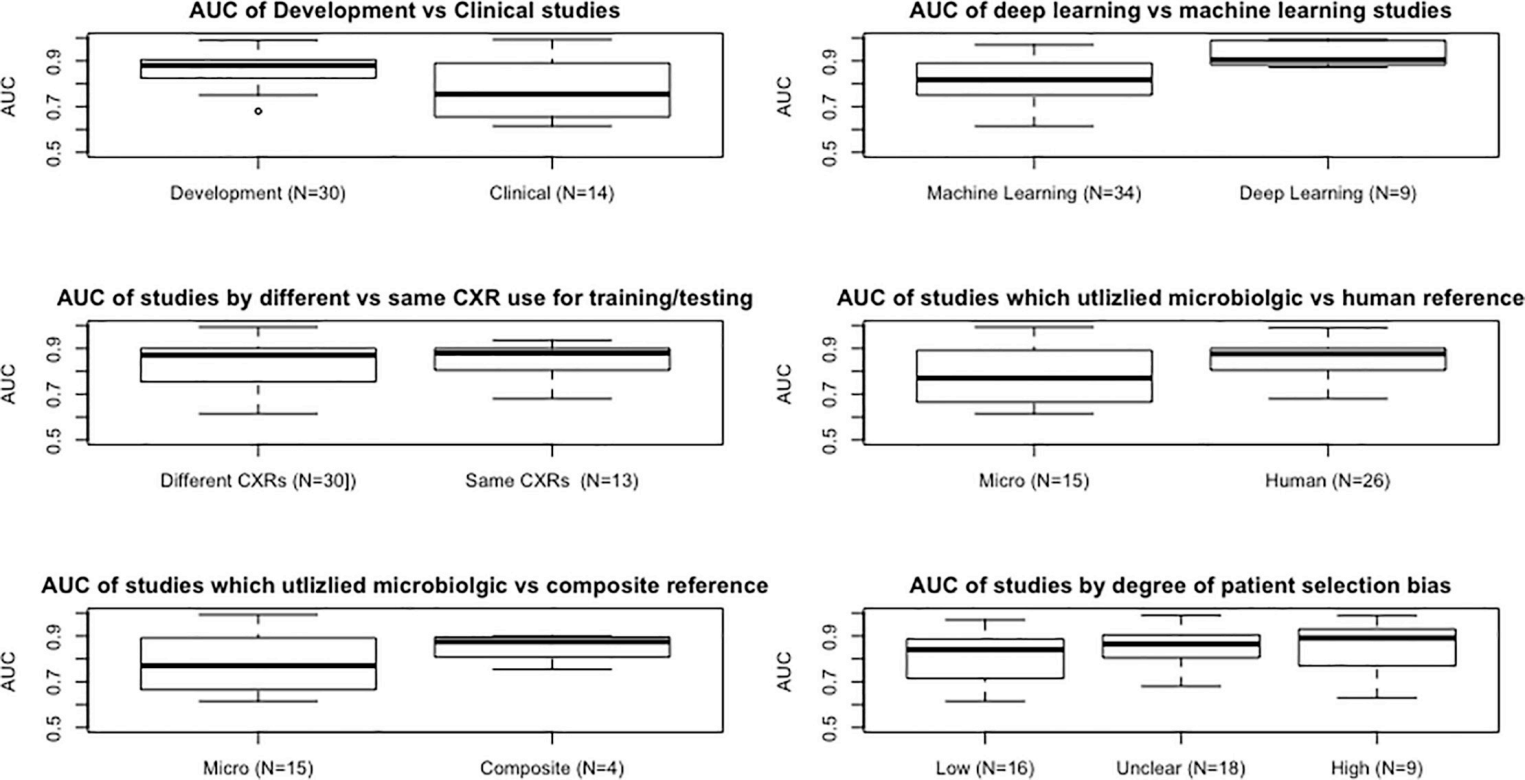
Boxplots of the AUC of studies stratified by software design, CXR usage, reference standard, and degree of patient selection, index test, and reference standard bias. AUC, area under the cure; Vs, versus; CXR, chest x-ray.

Fig 5 shows the distribution of reported AUCs stratified by study level characteristics. Reported AUCs were higher in: Development studies (median [IQR] AUC: 0.88 [0.82–0.90]) versus Clinical studies (0.75 [0.66–0.87]; p-value 0.004); and with DL (0.91 [0.88–0.99]) versus ML (0.82 [0.75–0.89]; *p* = 0.001). While not statistically significant, we found that the median AUC of studies using a human reader as the reference standard were higher than those studies using a microbiologic reference standard of 0.88 [0.81–0.90] versus 0.77 [0.67–0.89] respectively (*p* = 0.16). There was no significant difference in AUCs of studies that used the same CXRs as the source for software development and evaluation of diagnostic accuracy, or of the AUCs by the degree of patient selection, index test, or reference standard bias (Fig 5).

## Discussion

In this systematic review, we sought to determine the diagnostic accuracy of CAD software programs for detecting PTB on CXRs. Due to study heterogeneity, we did not meta-analyze the data. We identified a number of methodological limitations in the existing evidence base. Moreover, we identified a number of study-level factors associated with the reported accuracy, which should be taken into consideration when evaluating future CAD studies.

The majority of the CAD evidence base for PTB detection consists of Development studies. While many of these reported some measure of diagnostic accuracy, this was done without assessing the potential risks of bias arising from the databases that were used. Applying a widely accepted standardized tool—QUADAS-2—for evaluating the quality of diagnostic studies we found that the potential risk of bias was common in the databases used to evaluate CAD in Development studies. We suggest future development studies apply the QUADAS-2 tool to assess for bias of the databases (Box 1).

## Box 1. Recommendations for CAD accuracy study design elements

**Table:** 

**Recommendations for studies assessing CAD accuracy**
• For the databases used to assess CAD accuracy, describe whether CXR had been used for triage or screening purposes. • State whether results of the evaluation being reported are applicable to Triage or Screening CXR use-cases
• Apply QUADAS-2 to assess the risk of bias in the databases used to evaluate CAD’s diagnostic accuracy
• Describe how CXRs were selected for training and testing
• Use different CXRs from separate databases for training and testing
• Clearly define true positive PTB
• Use a microbiologic reference standard of culture (preferred) or NAAT
• For CAD that output a continuous score, preferably pre-specify the threshold used to differentiate between a positive and negative CAD result.
• For CAD that output a continuous score, report how the threshold score was determined
• State whether pre-training/verification of CAD with local CXRs is required prior to use in each setting

All Clinical studies evaluated the same commercially available software, CAD4TB. As noted above, meta-analysis was not completed due to the methodological heterogeneity, the lack of standardized threshold scores, and the variability of software versions used. While the software achieved high sensitivities (0.85 to 1.0), there was a large degree of variability in the reported specificities (0.23–0.69). Furthermore, the analysis in some studies was performed on CXRs from datasets or sites that may have also contributed to training the software, potentially resulting in an overestimation of the predictive power. Lastly, because the populations studied had very high HIV and TB prevalence, the results may have limited generalizability to other populations.

We identified a number of study-level factors that were associated with the reported AUC. These included the type of technology used to classify images, and whether it was a Development or Clinical study. The accuracy of DL vs ML studies was higher (median AUC DL vs ML p-value 0.001), suggesting superior diagnostic accuracy of DL technology. The median AUC of development studies was higher than clinical studies (p-value 0.004). This likely because of the greater risk of bias due to the lack of pre-specified threshold scores, the use of the same databases for training and testing, and the use of a human reader as the reference standard. Our findings also suggested that studies using a human reader reference standard may have systematically overestimated the diagnostic accuracy of CAD, as the median AUC of these studies was higher compared to studies that used a microbiologic reference; the differences were not statistically significant, however. We did not find a significant difference in AUCs from studies that used the same CXRs for training and testing. However, we can extrapolate from other studies that using the same databases for training and testing will results in the systematic overestimations of reported predative value [73].

We suggest some elements that could improve the clinical applicability of future studies of CAD. Studies should include a description of how CXRs were selected for training and testing. Furthermore, CXRs from distinct databases should be used for training and testing. Ideally, accuracy of CAD should be evaluated against a microbiologic reference standard. Lastly, if the software has a continuous output, the threshold score to differentiate between a positive or negative CXR should be reported, along with how this was determined (Box 1). The US Food and Drug Administration (FDA) requires all of these standards be met and additionally necessitates clear instructions for clinical use in their guidelines of CAD applied to radiology devices (17).

One potential weakness of this review is that we only included studies from the published literature, which could increase the risk that publication bias affected our reported results. Additionally, we restricted our search to English and French studies only. Furthermore, we were unable to complete a meta-analysis of the clinical studies and hence unable to comment on the pooled accuracy of CAD.

This systematic review highlights the need for additional research of CAD of PTB on CXR. To our knowledge, this is the first study to analyze the quality of current CXR databases that have been used to train and test multiple CAD software tools. We conclude that AI based CAD programs are promising, but more clinical studies are needed that minimize sources of potential bias to ensure validity of the findings outside of the study setting.

## Supporting information

S1 Appendix Search strategies. (PDF)

S2 Appendix Extraction form. (PDF)

S3 Appendix Prisma (Preferred reporting items for systematic reviews and meta-analyses) checklist. (DOCX)

S1 Table Demographics of CAD4TB studies with microbiologic reference standard. CAD, computer aided diagnosis; yrs, years; NR, not reported; TB, tuberculosis; HIV, human immunodeficiency virus
*This is the median, the mean age was not reported.
(PDF)

S2 Table Selection, enrolment of CAD4TB studies with microbiologic reference standard. NR, not reported; CAD, computer aided diagnosis; NAAT, nucleic acid amplification test
* Patients with an abnormal CXR as per radiologist reading, or presumptive TB based on TB symptoms received culture
** Patients with a normal CXR by CAD received an AFB smear, while patients with an abnormal CXR as per CAD received NAAT.
(PDF)

S3 Table Quality assessment of datasets used to test and train CAD software of development studies: Risk of bias and applicability concerns. AMPATH, Academic Model Providing Access to Healthcare; CH, Shenzhen Hospital, China; F&T, Find and Treat; IN, Indian collection New Delhi; JSRT, Japanese Society of Radiology; KIT, Korean Institute of Tuberculosis; MC, Montgomery County; YU AWHE, Yonsei University Annual Worker's health examination; SNUH, Seoul National University Hospital; TJH, Thomas Jefferson Hospital dataset; U, unclear; H, high; NA, not applicable; L, low
* Calgary dataset included preselected “typical PTB” images
** JSRT data set does not include PTB cases, but rather comprises images with single pulmonary nodules, confirmed by computed tomography and histology as either benign or pathologic.
(PDF)

S4 Table Quality assessment (QUADAS 2) summary of development studies: Risk of bias & applicability concerns. (PDF)

S5 Table Quality assessment (QUADAS 2) summary of clinical studies: Risk of bias and applicability concerns. (PDF)
