# Supplementary material for: A systematic review of the diagnostic accuracy of artificial intelligence-based computer programs to analyze chest x-rays for pulmonary tuberculosis
Source: PLoS One. 2019 Sep 3;14(9):e0221339. doi: 10.1371/journal.pone.0221339 (PMC6719854; doi:10.1371/journal.pone.0221339)
Supplement: S1 Appendix — (PDF) [file pone.0221339.s001.pdf]

Appendix

S1 Appendix. Search Strategies (2 searches completed)

1. Original Search

CAD SysRev-Meta analysis project

Search results summary

Last date searches ran = Jan 20, 2017

Name of the Database	Number of citations	Name of the search saved in the database
Medline (OVID)	1269	CAD_Medline_Search_Jan20
EMBASE (OVID)	1171	EMBASE_Search_Jan19
PubMed	961	CAD-SYSREV_Jan20_2017
Scopus	123	NA
Total	3524	
**Total after removing duplicated records**	**2360**	

Search Histories

Medline:

Ovid MEDLINE(R) and Epub Ahead of Print, In-Process & Other Non-Indexed Citations, Ovid MEDLINE(R) Daily <1946 to Present>			
**#**	**Search Statement**	**Results**	**Annotation**
1	*tuberculosis/ or tuberculosis, multidrug-resistant/ or tuberculosis, pulmonary/	154348	
2	pulmonary tuberculosis.ab,kf,ti.	33170	
3	pulmonary tuberculoses.ab,kf,ti.	18	

4	tuberculosis.ab,kf,ti.	210056	
5	TB.ab,kf,ti.	43005	
6	mycobacterium tuberculosis.ab,kf,ti.	47304	
7	Mycobacterium tuberculosis/	50082	
8	1 or 2 or 3 or 4 or 5 or 6 or 7	247839	
9	diagnosis, computer-assisted/ or image interpretation, computer-assisted/	67900	
10	decision making, computer-assisted/	3005	
11	*diagnostic imaging/ or *radiography/	45479	
12	(diagnostic adj2 (imaging or radiography)).ab,kf,ti.	15010	
13	"Image Processing, Computer-Assisted"/	124366	
14	medical imaging.ab,kf,ti.	7203	
15	diagnostic imaging.ab,kf,ti.	12687	
16	chest radiographs.ab,kf,ti.	7800	
17	chest radiography.ab,kf,ti.	7460	
18	chest imaging.ab,kf,ti.	859	
19	exp Machine Learning/	5823	
20	supervised learning.ab,kf,ti.	1692	
21	multiple-instance learning.ab,kf,ti.	95	
22	machine learning.ab,kf,ti.	11801	
23	lung segmentation*.ab,kf,ti.	158	
24	(image adj1 (interpretation or processing or analysis)).ab,kf,ti.	47972	
25	(computer* adj3 diagnosis).ab,kf,ti.	4047	

26	(computer* adj2 detection).ab,kf,ti.	1907	
27	(computer* adj2 screening).ab,kf,ti.	608	
28	(computer* adj2 interpretation).ab,kf,ti.	512	
29	(software adj2 (diagnosis or detection or screening or interpretation)).ab,kf,ti.	623	
30	(automated or automatic).ab,kf,ti.	161434	
31	or/9-30	453603	
32	exp "sensitivity and specificity"/	552842	
33	*diagnosis/ or *diagnosis, differential/ or exp diagnostic errors/	149258	
34	"sensitiv*".ab,kf,ti.	1308412	
35	specificity.ab,kf,ti.	440638	
36	di.fs.	2488090	
37	(screening adj2 (accuracy or accurate or correct or error or precise)).ab,kf,ti.	1788	
38	(diagnos* adj2 (accuracy or accurate or correct or error or precise)).ab,kf,ti.	94724	
39	exp "reproducibility of results"/	373630	
40	((validity or validation or reliability or reliable or predict*) adj3 (results or findings or screening or diagnos*)).ab,kf,ti.	89473	
41	Software Validation/	1735	
42	performance*.ab,kf,ti.	798744	
43	outperform*.ab,kf,ti.	23186	
44	comparable*.ab,kf,ti.	348653	
45	compar*.ab,kf,ti.	4882219	
46	chest X-ray*.ab,kf,ti.	22538	
47	or/32-46	8734531	

48	8 and 31 and 47	2220	
49	limit 48 to (english or french)	1925	
50	limit 49 to yr="2005 -Current"	1269	

EMBASE:

Embase <1996 to 2017 Week 03>			
**#**	**Search Statement**	**Results**	**Annotation**
1	tuberculosis/	93255	
2	lung tuberculosis/	29696	
3	pulmonary tuberculosis.ab,hw,kw,ti.	15248	
4	lung tuberculosis.ab,hw,kw,ti.	29781	
5	pulmonary tuberculoses.ab,hw,kw,ti.	5	
6	lung tuberculoses.ab,hw,kw,ti.	1	
7	TB.ab,hw,kw,ti.	46526	
8	Mycobacterium tuberculosis/	49630	
9	mycobacterium tuberculosis.ab,hw,kw,ti.	54655	
10	1 or 2 or 3 or 4 or 5 or 6 or 7 or 8 or 9	148529	
11	computer assisted diagnosis/ or computer assisted radiography/	31792	
12	exp *diagnostic imaging/	19878	
13	*radiography/ or computer assisted radiography/	13524	
14	(diagnostic adj2 (imaging or radiography)).ab,hw,kw,ti.	129205	
15	*image analysis/	8386	

16	computer heuristics/	43	
17	machine learning/ or online analytical processing/ or supervised machine learning/ or unsupervised machine learning/	11771	
18	supervised learning.ab,kw,ti.	1690	
19	multiple-instance learning.ab,kw,ti.	71	
20	machine learning.ab,kw,ti.	11539	
21	"lung segmentation*".ab,kw,ti.	178	
22	(image adj1 (interpretation or processing or analysis)).ab,kw,ti.	47593	
23	(software adj2 (diagnosis or detection or screening or interpretation)).ab,kw,ti.	865	
24	(automated or automatic).ab,kw,ti.	152273	
25	automated pattern recognition/	14666	
26	(computer adj2 diagnosis).ab,kw,ti.	3114	
27	(computer adj2 detection).ab,kw,ti.	1659	
28	(computer adj2 screening).ab,kw,ti.	327	
29	(computer adj2 interpretation).ab,kw,ti.	243	
30	or/11-29	377492	
31	"sensitivity and specificity"/	267362	
32	*sensitivity analysis/	2037	
33	differential diagnosis/	243015	
34	exp diagnostic error/	69447	
35	di.fs.	2079273	

36	"sensitiv*".ab,kw,ti.	1089591	
37	specificity.ab,kw,ti.	381205	
38	(screening adj2 (accuracy or accurate or correct or error or precise)).ab,kw,ti.	1831	
39	(diagnos* adj2 (accuracy or accurate or correct or error or precise)).ab,kw,ti.	97471	
40	*reproducibility/	7960	
41	((validity or validation or reliability or reliable or predict*) adj3 (results or findings or screening or diagnos*)).ab,kw,ti.	107267	
42	software validation/	20	
43	"performance*".ab,kw,ti.	717592	
44	"outperform*".ab,kw,ti.	20363	
45	"compar*".ab,kw,ti.	4558625	
46	or/31-45	7531693	
47	10 and 30 and 46	1997	
48	limit 47 to ((english or french) and yr="2005 -Current")	1250	
49	remove duplicates from 48	1171	

PubMed:

(((((((chest radiographs[Title/Abstract] OR chest radiography[Title/Abstract] OR chest imaging[Title/Abstract] OR chest X-ray*[Title/Abstract]))) OR ((sensitiv*[Title/Abstract] OR sensitivity and specificity[MeSH Terms] OR diagnose[Title/Abstract] OR diagnosed[Title/Abstract] OR diagnoses[Title/Abstract] OR diagnosing[Title/Abstract] OR diagnosis[Title/Abstract] OR diagnostic[Title/Abstract] OR diagnosis[MeSH:noexp] OR diagnostic *[MeSH:noexp] OR diagnosis, differential[MeSH:noexp] OR diagnosis[Subheading:noexp] OR "Reproducibility of Results"[Mesh] OR "Software Validation"[Mesh] OR "Diagnostic Imaging"[Majr:noexp] OR "diagnostic imaging"[Subheading] OR "Radiography"[Majr:noexp] OR "methods"[Subheading])))) AND (("Diagnosis, Computer-Assisted"[Mesh] OR "Decision Making, Computer-Assisted"[Mesh:noexp] OR "Image Processing, Computer-Assisted"[Mesh] OR "Machine Learning"[Mesh] OR computer[tiab] OR computer[ot] OR software[tiab] OR software[ot] OR automated[tiab] OR automatic[tiab] OR

automated[ot] OR automatic[ot]))) AND ("Tuberculosis"[Majr:noexp] OR "Tuberculosis, Pulmonary"[Mesh] OR "Mycobacterium tuberculosis"[Mesh] OR tuberculosis[tiab] OR tuberculosis[ot] OR "pulmonary tuberculoses"[tiab] OR TB[tiab] OR TB[ot] OR "mycobacterium tuberculosis"[tiab] OR "mycobacterium tuberculosis"[ot]))) NOT ("Animals"[Mesh] NOT "Humans"[Mesh])

Scopus:

(TITLE-ABS-KEY (chest radiograph* OR chest imaging OR chest x-ray* OR medical imaging OR diagnos*)) AND ((TITLE-ABS-KEY ("machine learning" OR "supervised learning" OR automated OR automatic OR "lung segmentation*") OR TITLE-ABS-KEY (image W/2 (diagnos* OR interpretation OR processing OR analysis)) OR TITLE-ABS-KEY (computer W/2 (diagnos* OR detection OR screening OR interpretation)) OR TITLE-ABS-KEY (software W/2 (diagnos* OR detection OR screening OR interpretation)))) AND (TITLE-ABS-KEY (tuberculosis OR tb) AND PUBYEAR > 2004) AND (LIMIT-TO (LANGUAGE , "English") OR LIMIT-TO (LANGUAGE , "French"))

2. Updated Search

CAD SysRev-Meta analysis project – Search updates

Search results summary for updates

Last date searches ran = November 28, 2017

**Name of the Database**	**Number of citations**	**Name of the search saved in the database**
Medline (OVID)	143 records	CAD_Medline_Search_Jan20
EMBASE (OVID)	150 records	EMBASE_Search_Jan19
PubMed	142 records	CAD-SYSREV_Jan20_2017
Scopus	18 records	NA
Total	453 records	
**Total after removing duplicated records**	**337 records**	

Database: Ovid MEDLINE(R) and Epub Ahead of Print, In-Process & Other Non-Indexed Citations, Ovid MEDLINE(R) Daily <1946 to Present>

Search Strategy:

----

1 *tuberculosis/ or tuberculosis, multidrug-resistant/ or tuberculosis, pulmonary/ (146959)

2 pulmonary tuberculosis.ab,kf,ti. (32026)

3 pulmonary tuberculoses.ab,kf,ti. (15)

4 tuberculosis.ab,kf,ti. (203835)

5 TB.ab,kf,ti. (44194)

6 mycobacterium tuberculosis.ab,kf,ti. (45263)

7 Mycobacterium tuberculosis/ (47471)

8 1 or 2 or 3 or 4 or 5 or 6 or 7 (240017)

9 diagnosis, computer-assisted/ or image interpretation, computer-assisted/ (66091)

10 decision making, computer-assisted/ (2870)

11 *diagnostic imaging/ or *radiography/ (44230)

12 (diagnostic adj2 (imaging or radiography)).ab,kf,ti. (15455)

13 "Image Processing, Computer-Assisted"/ (120667)

14 medical imaging.ab,kf,ti. (7842)

15 diagnostic imaging.ab,kf,ti. (13019)

16 chest radiographs.ab,kf,ti. (7110)

17 chest radiography.ab,kf,ti. (7200)

18 chest imaging.ab,kf,ti. (907)

19 exp Machine Learning/ (7405)

20 supervised learning.ab,kf,ti. (1736)

21 multiple-instance learning.ab,kf,ti. (98)

22 machine learning.ab,kf,ti. (13401)

23 lung segmentation*.ab,kf,ti. (161)

24 (image adj1 (interpretation or processing or analysis)).ab,kf,ti. (48161)

25 (computer* adj3 diagnosis).ab,kf,ti. (4126)

26 (computer* adj2 detection).ab,kf,ti. (1952)

27 (computer* adj2 screening).ab,kf,ti. (581)

28 (computer* adj2 interpretation).ab,kf,ti. (513)

29 (software adj2 (diagnosis or detection or screening or interpretation)).ab,kf,ti. (634)

30 (automated or automatic).ab,kf,ti. (164147)

31 or/9-30 (451614)

32 exp "sensitivity and specificity"/ (559273)

33 *diagnosis/ or *diagnosis, differential/ or exp diagnostic errors/ (146685)

34 "sensitiv*".ab,kf,ti. (1296376)

35 specificity.ab,kf,ti. (433249)

36 di.fs. (2498848)

37 (screening adj2 (accuracy or accurate or correct or error or precise)).ab,kf,ti. (1790)

38 (diagnos* adj2 (accuracy or accurate or correct or error or precise)).ab,kf,ti. (97918)

39 exp "reproducibility of results"/ (379399)

40 ((validity or validation or reliability or reliable or predict*) adj3 (results or findings or screening or diagnos*)).ab,kf,ti. (91509)

41 Software Validation/ (1675)

42 performance*.ab,kf,ti. (830516)

43 outperform*.ab,kf,ti. (24724)

44 comparable*.ab,kf,ti. (352640)

45 compar*.ab,kf,ti. (4948322)

46 chest X-ray*.ab,kf,ti. (21656)

47 or/32-46 (8793187)

48 8 and 31 and 47 (2173)

49 limit 48 to (english or french) (1900)

50 limit 49 to yr="2005 -Current" (1280)

51 (2017012* or 201702* or 201703* or 201704* or 201705* or 201706* or 201707* or 201708* or "201709" or 20171*).dc,ed. (1587377)

52 50 and 51 (143)

Database: Embase <1996 to 2017 Week 48>

Search Strategy:

----

1 tuberculosis/ (75418)

2 lung tuberculosis/ (29608)

3 pulmonary tuberculosis.ab,hw,kw,ti. (15835)

4 lung tuberculosis.ab,hw,kw,ti. (29711)

5 pulmonary tuberculoses.ab,hw,kw,ti. (5)

6 lung tuberculoses.ab,hw,kw,ti. (1)

7 TB.ab,hw,kw,ti. (51757)

8 Mycobacterium tuberculosis/ (49023)

9 mycobacterium tuberculosis.ab,hw,kw,ti. (57432)

10 1 or 2 or 3 or 4 or 5 or 6 or 7 or 8 or 9 (148283)

11 computer assisted diagnosis/ or computer assisted radiography/ (32691)

12 exp *diagnostic imaging/ (24719)

13 *radiography/ or computer assisted radiography/ (9783)

14 (diagnostic adj2 (imaging or radiography)).ab,hw,kw,ti. (144128)

15 *image analysis/ (8104)

16 computer heuristics/ (111)

17 machine learning/ or online analytical processing/ or supervised machine learning/ or unsupervised machine learning/ (13437)

18 supervised learning.ab,kw,ti. (1940)

19 multiple-instance learning.ab,kw,ti. (84)

20 machine learning.ab,kw,ti. (14958)

21 "lung segmentation*".ab,kw,ti. (213)

22 (image adj1 (interpretation or processing or analysis)).ab,kw,ti. (53111)

23 (software adj2 (diagnosis or detection or screening or interpretation)).ab,kw,ti. (955)

24 (automated or automatic).ab,kw,ti. (169290)

25 automated pattern recognition/ (15200)

26 (computer adj2 diagnosis).ab,kw,ti. (3632)

27 (computer adj2 detection).ab,kw,ti. (1893)

28 (computer adj2 screening).ab,kw,ti. (358)

29 (computer adj2 interpretation).ab,kw,ti. (267)

30 or/11-29 (416263)

31 "sensitivity and specificity"/ (283617)

32 *sensitivity analysis/ (1415)

33 differential diagnosis/ (240445)

34 exp diagnostic error/ (70488)

35 di.fs. (2205767)

36 "sensitiv*".ab,kw,ti. (1179496)

37 specificity.ab,kw,ti. (414140)

38 (screening adj2 (accuracy or accurate or correct or error or precise)).ab,kw,ti. (2012)

39 (diagnos* adj2 (accuracy or accurate or correct or error or precise)).ab,kw,ti. (107467)

40 *reproducibility/ (3873)

41 ((validity or validation or reliability or reliable or predict*) adj3 (results or findings or screening or diagnos*)).ab,kw,ti. (119311)

42 software validation/ (87)

43 "performance*".ab,kw,ti. (794603)

44 "outperform*".ab,kw,ti. (24014)

45 "compar*".ab,kw,ti. (4997029)

46 or/31-45 (8165931)

47 10 and 30 and 46 (2036)

48 limit 47 to ((english or french) and yr="2005 -Current") (1328)

49 remove duplicates from 48 (1255)

50 limit 49 to dd=20170120-20171128 (150)

Scopus update, November 28, 2017

18 records

(TITLE-ABS-KEY (chest AND radiograph* OR chest AND imaging OR chest AND x-ray* OR medical AND imaging OR diagnos*)) AND ((TITLE-ABS-KEY ("machine learning" OR "supervised learning" OR automated OR automatic OR "lung segmentation*") OR TITLE-ABS-KEY (image W/2 (diagnos* OR interpretation OR processing OR analysis)) OR TITLE-ABS-KEY (computer W/2 (diagnos* OR detection OR screening OR interpretation)) OR TITLE-ABS-KEY (software W/2 (diagnos* OR detection OR screening OR interpretation)))) AND (TITLE-ABS-KEY (tuberculosis OR tb) AND PUBYEAR > 2004) AND ORIG-LOAD-DATE AFT 20170120 AND (LIMIT-TO (LANGUAGE , "English") OR LIMIT-TO (LANGUAGE , "French"))

Recent queries in pubmed

Search	Query	Items found	Time

#2	Search (((((((((chest radiographs[Title/Abstract] OR chest radiography[Title/Abstract] OR chest imaging[Title/Abstract] OR chest X-ray*[Title/Abstract]))) OR ((sensitiv*[Title/Abstract] OR sensitivity and specificity[MeSH Terms] OR diagnose[Title/Abstract] OR diagnosed[Title/Abstract] OR diagnoses[Title/Abstract] OR diagnosing[Title/Abstract] OR diagnosis[Title/Abstract] OR diagnostic[Title/Abstract] OR diagnosis[MeSH:noexp] OR diagnostic *[MeSH:noexp] OR diagnosis, differential[MeSH:noexp] OR diagnosis[Subheading:noexp] OR "Reproducibility of Results"[Mesh] OR "Software Validation"[Mesh] OR "Diagnostic Imaging"[Majr:noexp] OR "diagnostic imaging"[Subheading] OR "Radiography"[Majr:noexp] OR "methods"[Subheading])))) AND (("Diagnosis, Computer-Assisted"[Mesh] OR "Decision Making, Computer-Assisted"[Mesh:noexp] OR "Image Processing, Computer-Assisted"[Mesh] OR "Machine Learning"[Mesh] OR computer[tiab] OR computer[ot] OR software[tiab] OR software[ot] OR automated[tiab] OR automatic[tiab] OR automated[ot] OR automatic[ot]))) AND ("Tuberculosis"[Majr:noexp] OR "Tuberculosis, Pulmonary"[Mesh] OR "Mycobacterium tuberculosis"[Mesh] OR tuberculosis[tiab] OR tuberculosis[ot] OR "pulmonary tuberculoses"[tiab] OR TB[tiab] OR TB[ot] OR "mycobacterium tuberculosis"[tiab] OR "mycobacterium tuberculosis"[ot]))) NOT ("Animals"[Mesh] NOT "Humans"[Mesh]) AND (("2005/01/01"[PDat] : "3000/12/31"[PDat]) AND (French[lang] OR English[lang]))) AND "2017/01/20 15.00"[MHDA]:"2017/11/28 15.00"[MHDA]	142	9:30:24

3. New Updated Search

CAD SysRev-Meta analysis project – Search updates
Search results summary for updates

Last date searches ran = February 13, 2019

Name of the Database	Number of citations	Name of the search saved in the database
Medline (OVID)	227 records	CAD_Medline_Search_Feb13-19
EMBASE (OVID)	260 records	EMBASE_Search_Jan19
PubMed	211 records	CAD-SYSREV_Jan20_2017 "2017/11/28"[MHDA]:"2019/02/13"[MHDA] OR "2017
Scopus	37 records	NA
Total	735 records	
**Total after removing duplicated records**	**515 records**	

Database: Ovid MEDLINE(R) and Epub Ahead of Print, In-Process & Other Non-Indexed Citations, Ovid MEDLINE(R) Daily <1946 to Present>

Search Strategy:

----

1 *tuberculosis/ or tuberculosis, multidrug-resistant/ or tuberculosis, pulmonary/ (144914)

2 pulmonary tuberculoses.ab,kf,ti. (15)

3 tuberculosis.ab,kf,ti. (203032)

4 TB.ab,kf,ti. (48048)

5 Mycobacterium tuberculosis/ (47029)

6 1 or 2 or 3 or 4 or 5 (241361)

7 diagnosis, computer-assisted/ or image interpretation, computer-assisted/ (63451)

8 decision making, computer-assisted/ (2682)

9 *diagnostic imaging/ or *radiography/ (42652)

10 (diagnostic adj2 (imaging or radiography)).ab,kf,ti. (15807)

11 "Image Processing, Computer-Assisted"/ (115743)

12 medical imaging.ab,kf,ti. (7908)

13 diagnostic imaging.ab,kf,ti. (13391)

14 chest radiographs.ab,kf,ti. (6751)

15 chest radiography.ab,kf,ti. (7053)

16 chest imaging.ab,kf,ti. (929)

17 exp Machine Learning/ (10228)

18 supervised learning.ab,kf,ti. (1971)

19 multiple-instance learning.ab,kf,ti. (108)

20 machine learning.ab,kf,ti. (18297)

21 lung segmentation*.ab,kf,ti. (152)

22 (image adj1 (interpretation or processing or analysis)).ab,kf,ti. (47938)

23 (computer* adj3 diagnosis).ab,kf,ti. (4161)

24 (computer* adj2 detection).ab,kf,ti. (1828)

25 (computer* adj2 screening).ab,kf,ti. (564)

26 (computer* adj2 interpretation).ab,kf,ti. (491)

27 (software adj2 (diagnosis or detection or screening or interpretation)).ab,kf,ti. (651)

28 (automated or automatic).ab,kf,ti. (164609)

29 or/7-28 (448434)

30 exp "sensitivity and specificity"/ (544799)

31 *diagnosis/ or *diagnosis, differential/ or exp diagnostic errors/ (140360)

32 "sensitiv*".ab,kf,ti. (1282201)

33 specificity.ab,kf,ti. (428044)

34 di.fs. (2418102)

35 (screening adj2 (accuracy or accurate or correct or error or precise)).ab,kf,ti. (1866)

36 (diagnos* adj2 (accuracy or accurate or correct or error or precise)).ab,kf,ti. (98423)

37 exp "reproducibility of results"/ (372014)

38 ((validity or validation or reliability or reliable or predict*) adj3 (results or findings or screening or diagnos*)).ab,kf,ti. (93040)

39 Software Validation/ (1558)

40 performance*.ab,kf,ti. (858525)

41 outperform*.ab,kf,ti. (27900)

42 compar*.ab,kf,ti. (4917296)

43 chest X-ray*.ab,kf,ti. (21322)

44 or/30-43 (8692147)

45 6 and 29 and 44 (2213)

46 limit 45 to (english or french) (1925)

47 limit 46 to yr="2005 -Current" (1331)

48 ("20171128" or "20171129" or 2017113* or 201712* or 2018* or 2019*).dt,ed,ez. (2347749)

49 47 and 48 (227)

****

Database: Embase Classic+Embase <1947 to 2019 February 12>

Search Strategy:

----

1 tuberculosis/ (146966)

2 lung tuberculosis/ (73157)

3 pulmonary tuberculosis.ab,hw,kw,ti. (37656)

4 lung tuberculosis.ab,hw,kw,ti. (73386)

5 pulmonary tuberculoses.ab,hw,kw,ti. (16)

6 lung tuberculoses.ab,hw,kw,ti. (1)

7 TB.ab,hw,kw,ti. (84852)

8 Mycobacterium tuberculosis/ (71644)

9 mycobacterium tuberculosis.ab,hw,kw,ti. (83083)

10 1 or 2 or 3 or 4 or 5 or 6 or 7 or 8 or 9 (289226)

11 computer assisted diagnosis/ or computer assisted radiography/ (40330)

12 exp *diagnostic imaging/ (37927)

13 *radiography/ or computer assisted radiography/ (26287)

14 (diagnostic adj2 (imaging or radiography)).ab,hw,kw,ti. (178031)

15 *image analysis/ (10863)

16 computer heuristics/ (135)

17 machine learning/ or online analytical processing/ or supervised machine learning/ or unsupervised machine learning/ (19890)

18 supervised learning.ab,kw,ti. (2342)

19 multiple-instance learning.ab,kw,ti. (98)

20 machine learning.ab,kw,ti. (22040)

21 "lung segmentation*".ab,kw,ti. (235)

22 (image adj1 (interpretation or processing or analysis)).ab,kw,ti. (66308)

23 (software adj2 (diagnosis or detection or screening or interpretation)).ab,kw,ti. (1111)

24 (automated or automatic).ab,kw,ti. (230067)

25 automated pattern recognition/ (16209)

26 (computer adj2 diagnosis).ab,kw,ti. (4941)

27 (computer adj2 detection).ab,kw,ti. (2080)

28 (computer adj2 screening).ab,kw,ti. (439)

29 (computer adj2 interpretation).ab,kw,ti. (493)

30 or/11-29 (553754)

31 "sensitivity and specificity"/ (317572)

32 *sensitivity analysis/ (1660)

33 differential diagnosis/ (374329)

34 exp diagnostic error/ (90943)

35 di.fs. (3094823)

36 "sensitiv*".ab,kw,ti. (1657027)

37 specificity.ab,kw,ti. (563683)

38 (screening adj2 (accuracy or accurate or correct or error or precise)).ab,kw,ti. (2486)

39 (diagnos* adj2 (accuracy or accurate or correct or error or precise)).ab,kw,ti. (147396)

40 *reproducibility/ (5468)

41 ((validity or validation or reliability or reliable or predict*) adj3 (results or findings or screening or diagnos*)).ab,kw,ti. (149047)

42 software validation/ (155)

43 "performance*".ab,kw,ti. (1045323)

44 "outperform*".ab,kw,ti. (29399)

45 "compar*".ab,kw,ti. (6701429)

46 or/31-45 (11300687)

47 10 and 30 and 46 (2431)

48 limit 47 to ((english or french) and yr="2005 -Current") (1540)

49 limit 48 to dc=20171128-20190213 (264)

50 remove duplicates from 49 (260)

****

Scopus update, February 13, 2019

37 records

(TITLE-ABS-KEY (chest radiograph* OR chest imaging OR chest x-ray* OR medical imaging OR diagnos*)) AND ((TITLE-ABS-KEY ("machine learning" OR "supervised learning" OR automated OR automatic OR "lung segmentation*") OR TITLE-ABS-KEY (image W/2 (diagnos* OR interpretation OR processing OR analysis)) OR TITLE-ABS-KEY (computer W/2 (diagnos* OR detection OR screening OR interpretation)) OR TITLE-ABS-KEY (software W/2 (diagnos* OR detection OR screening OR interpretation)))) AND (TITLE-ABS-KEY (tuberculosis OR tb) AND PUBYEAR > 2004) AND (LIMIT-TO (LANGUAGE , "English") OR LIMIT-TO (LANGUAGE , "French")) AND ORIG-LOAD-DATE > 20171127

PubMed update, February 13, 2019

211 records

((((((((chest radiographs[Title/Abstract] OR chest radiography[Title/Abstract] OR chest imaging[Title/Abstract] OR chest X-ray*[Title/Abstract]))) OR ((sensitiv*[Title/Abstract] OR sensitivity and specificity[MeSH Terms] OR diagnose[Title/Abstract] OR diagnosed[Title/Abstract] OR diagnoses[Title/Abstract] OR diagnosing[Title/Abstract] OR diagnosis[Title/Abstract] OR diagnostic[Title/Abstract] OR diagnosis[MeSH:noexp] OR diagnostic *[MeSH:noexp] OR diagnosis, differential[MeSH:noexp] OR diagnosis[Subheading:noexp] OR "Reproducibility of Results"[Mesh] OR "Software Validation"[Mesh] OR "Diagnostic Imaging"[Majr:noexp] OR "diagnostic imaging"[Subheading] OR "Radiography"[Majr:noexp] OR "methods"[Subheading])))) AND (("Diagnosis, Computer-Assisted"[Mesh] OR "Decision Making, Computer-Assisted"[Mesh:noexp] OR "Image Processing, Computer-Assisted"[Mesh] OR "Machine Learning"[Mesh] OR computer[tiab] OR computer[ot] OR software[tiab] OR software[ot] OR automated[tiab] OR automatic[tiab] OR automated[ot] OR automatic[ot]))) AND ("Tuberculosis"[Majr:noexp] OR "Tuberculosis, Pulmonary"[Mesh] OR "Mycobacterium tuberculosis"[Mesh] OR tuberculosis[tiab] OR tuberculosis[ot] OR "pulmonary tuberculoses"[tiab] OR TB[tiab] OR TB[ot] OR "mycobacterium tuberculosis"[tiab] OR "mycobacterium tuberculosis"[ot]))) NOT ("Animals"[Mesh] NOT "Humans"[Mesh]) AND (("2005/01/01"[PDat] : "3000/12/31"[PDat]) AND (French[lang] OR English[lang]))) AND (("2017/01/20"[Date - Create] : "2017/11/28"[Date - Create] NOT "2017/01/20"[MHDA] : "2017/11/28"[MHDA]) OR "2017/11/28"[Date - Create] : "3000"[Date - Create] OR "2017/11/28"[MHDA] : "3000"[MHDA])
