# Supplementary material for: A systematic review of the diagnostic accuracy of artificial intelligence-based computer programs to analyze chest x-rays for pulmonary tuberculosis
Source: PLoS One. 2019 Sep 3;14(9):e0221339. doi: 10.1371/journal.pone.0221339 (PMC6719854; doi:10.1371/journal.pone.0221339)
Supplement: S2 Appendix — (PDF) [file pone.0221339.s002.pdf]

Extraction Form

1. COVER SHEET

* Required

1. **1.1. Study title:** *

____

2. **1.2. PI:** *

____

3. **1.2.1.Data extracted by:** *

____

4. **1.3. Contact address of data extractor:** *

____

5. **1.4.1.Month/Year (start of enrollment):**

____
Example: December 15, 2012

6. **1.4.2.Month/Year (end of enrollment):**

____
Example: December 15, 2012

7. **1.4.3.Final status of the study:** *
Mark only one oval.

- ◯ 1 Completed
- ◯ 2 Ongoing

8. **1.5. Type of publication:** *
Mark only one oval.

- ◯ 1 Full paper
- ◯ 2 Abstract
- ◯ 3 Letter
- ◯ 4 Unpublished data
- ◯ Other: ________________________________

9. **1.6. Funding source conflict of interest: study funded or undertaken by company/proprietary owner of software or x-ray machine; kits supplied by company (1=yes, 2=no).** *
Mark only one oval.

- ◯ 1
- ◯ 2

10. **1.6.1.If 1, specify the support**

2. If excluded from quantitative fill the following section,Information on exclusion of study

11. **2.1. Reason for exclusion**
Mark only one oval.

- 1 Not a CAD study
- 2 CAD with no microbiological reference
- 3 Technical study only
- Screening CAD4TB study

3. DATA ON SOFTWARE AND X-RAY EQUIPMENT

Information on software and x-ray

12. **3.1. Name of software**

13. **3.2. What was the version number?**

14. **3.3. What type of software was being used?**
Mark only one oval.

- 1 Machine learning
- 2 Deep learning

15. **3.4. What type of health care facility was the x-ray taken for training? choose all that apply**
Check all that apply.

- [ ] 1 Primary Health clinics
- [ ] 2 National/provincial/state/municipal TB Program Clinic
- [ ] 3 Reference center (eg. Secondary/tertiary clinic or hospital)
- [ ] 4 Mobile diagnostic unit
- [ ] 5 Public Hospital
- [ ] 6 Private Hospital
- [ ] 7 Public Laboratories
- [ ] 8 Private Laboratories

16. **3.5. What type of health care facility was the x-ray taken for evaluation? choose all that apply**
Check all that apply.

- [ ] 1 Primary Health clinics
- [ ] 2 National/provincial/state/municipal TB Program Clinic
- [ ] 3 Reference center (eg. Secondary/tertiary clinic or hospital)
- [ ] 4 Mobile diagnostic unit
- [ ] 5 Public Hospital
- [ ] 6 Private Hospital
- [ ] 7 Public Laboratories
- [ ] 8 Private Laboratories

17. **3.6. What was the make and model of the X-ray machine used? (Please name all if different ones were used)**

____
____
____
____
____

18. **3.7. How were the digital x-ray generated? (Select all that apply)**
Check all that apply.

- [ ] 1 Physical films were scanned
- [ ] 2 Computed Radiography (CR)
- [ ] 3 Direct Digital Radiography (DR, or DDR)

19. **3.8. How were the x-rays read by the human readers? (Select all that apply)**
Check all that apply.

- [ ] 1 X-ray film read (physical film)
- [ ] 2 Digital images were viewed on screen

4.STUDY DESIGN

20. **4.1. Country or countries where x-rays were performed. Please list all:**

____
____
____
____
____

21. **4.2. What was the overall purpose of the study?**
Mark only one oval.

- 1 Assessing the diagnostic accuracy of CAD
- 2 Technical report on development and performance in a diagnostic algorithm..
- 3 Cost or cost effectiveness related analysis
- Other: ______

22. **4.2.1. If it was a technical report , number of databases used to train CAD:**

______

23. **4.2.2. If it was a technical report, Number of CXRs used to train CAD:**

______

24. **4.2.3. If it was a technical report, Where were the databases from:**

______

25. **4.3. For what purpose was CXR being used in the study?**
Mark only one oval.

- 1 TB screening (active case finding)
- 2 TB triage (patients with active TB symptoms)
- 3 Prevalence survey
- 4 Visual aid to human reader for analyzing CXR
- Other: ______

26. **4.4. CXR performance and data collection (select one):**
Mark only one oval.

- 1 The CXR was prospectively performed and collected data for this study
- 2 The CXR and other data had been previously collected as part of another study
- 3 CXR and data was collected from previously performed investigations for routine patient management

4. Domain 1: Patient Selection

27. **4.5. Answer only If the study used data that were previously collected: Were all participants from the original study used for the study? (1=yes, 2=no)**
Mark only one oval.

- 1
- 2

28. **4.5.1. If 2, How were participants selected for inclusion in the study? Explain:**

29. **4.6. Was the sample size estimated prior to the start of the study? (1=yes, 2=no, 888=not reported)**
Mark only one oval.
- 1
- 2
- 888

30. **4.6.1. If 1, what was the sample size?**

31. **4.7. What was the enrolled sample size?**
Number =

32. **4.8. What sampling method was used to select patients?**
Mark only one oval.
- 1 Random sample
- 2 Purposive sampling
- 3 Quota sampling
- 4 Consecutive enrollment
- 5 Case control sampling
- 6 Not reported
- Other:

4. Inclusion/Exclusion Criteria

33. **4.9. What were the inclusion/exclusion criteria?**
Mark only one oval.
- Not applicable: our study did not have inclusion/exclusion criteria (go to Q 4.12)

34. **4.9.1. Inclusion criteria:**

35. **4.9.2. Exclusion criteria:**

36. **4.10. Were all persons enrolled suspected to have pulmonary TB (PTB)? (1=yes, 2=no, 888=not reported)**
Mark only one oval.

- 1
- 2
- 888

37. **4.10.1. If 1, Please explain how this was defined**

38. **4.11. Were asymptomatic patients eligible for enrollment? (1=yes, 2=no)**
Mark only one oval.

- 1
- 2

39. **4.12. Were people living with HIV eligible for enrollment? (1=yes, 2=no)**
Mark only one oval.

- 1
- 2

40. **4.12.1. if 1, number**

41. **4.13. Were patients with PMH (past medical history) of TB eligible for enrollment? (1=yes, 2=no)**
Mark only one oval.

- 1
- 2

42. **4.13.1. if 1, number**

____

43. **4.14. Were pregnant women eligible for enrolment? (1=yes, 2=no)**
Mark only one oval.

- 1
- 2

44. **4.14.1. if 1, number**

____

45. **4.15. Were children (age <15) eligible for enrollment? (1=yes, 2=no)**
Mark only one oval.

- 1
- 2

46. **4.15.1. if 1, number**

____

Skip to question 47.

4. QUADAS Domain 1: Questions

Risk of bias

47. **4.16. Describe methods of patient selection (one to two sentences)**

____
____
____
____
____

48. **4.17. Was a consecutive or random sample of patients enrolled? (1= yes a random sample was used, 2=consecutive sample used, 3=unclear)**
Mark only one oval.

- 1
- 2
- 999

49. **4.18. Was a case-control design avoided? (1=yes, 2=no, 999=unclear)**
Mark only one oval.

- 1
- 2
- 999

50. **4.19. Did the study avoid inappropriate inappropriate exclusions? (1=yes, 2=no, 999=unclear)**
Mark only one oval.

- 1
- 2
- 999

51. **4.20. Could the selection of patients have introduced bias? (3=low, 4=high, 999=unclear)**
Mark only one oval.

- 3
- 4
- 999

4. QUADAS Domain 1: Questions

Concerns regarding applicability

52. **4.21. Describe included patients (prior testing, presentation, intended use of index test and setting) in one to two sentences.**

____

53. **4.22. Is there concern that the included patients do not match the review question? (3=low, 4=high, 999=unclear)**
Mark only one oval.

- 3
- 4
- 999

5. Domain 2: Index test

54. **5.1. Methodology for abnormality scoring by humans**
Mark only one oval.

- 1 Based on specific categories
- 2 Based on a scoring algorithm
- Other: ______________________________

55. **5.2. What abnormality scoring algorithm or categories were used by the readers? (if applicable)**

____

56. **5.3. Did readers rate the quality of the X-rays? (1=yes, 2=no)**
Mark only one oval.

- ( ) 1
- ( ) 2

57. **5.4. Was interpretation categorical or continuous or both?**
Mark only one oval.

- ( ) 1 Categorical
- ( ) 2 Continuous
- ( ) 3 Both

58. **5.4.1. If categorical,**
Mark only one oval.

- ( ) normal vs. abnormal
- ( ) Other: ________________________

59. **5.4.2. If continuous, what was the scale used?**

____

60. **5.5. Were the criteria to distinguish between positive (abnormal) and negative (normal) chest x-rays pre- specified? (1=yes, 2=no)**
Mark only one oval.

- ( ) 1
- ( ) 2

61. **5.6. If a categorical analysis was done for the software, how was the cutoff for software determined?**
Mark only one oval.

- ( ) 1 Using previous collected CXR data of population to generate ROC curve
- ( ) 2 ROC curve was used to identify threshold score that achieved same specificity as field officer
- ( ) 3 Arbitrary cutoff
- ( ) 4 Not reported

62. **5.7. What were the cutoffs for software?**
Number

____

63. **5.8. If continuous scores were used for software, was AUC curve documented? (1=yes, 2=no)**
Mark only one oval.

- 1
- 2

64. **5.9. Was diagnostic heterogeneity assessed? (1=yes, 2=no, 888=not reported)**
Mark only one oval.

- 1
- 2
- 888

65. **5.9.1. If 1, what groups were assessed? check all that apply**
Check all that apply.

- [ ] 1 smear positive
- [ ] 2 smear negative
- [ ] 3 PLWH
- [ ] 4 women
- [ ] 5 children
- [ ] Other: ____________________

5. QUADAS Domain 2: Questions

Risk of bias

66. **5.10. Briefly describe the index test and how it was conducted and interpreted in one to two sentences.**

____

67. **5.11. Were the CAD results interpreted without the knowledge of the results of the reference standard? (1=yes, 2=no, unclear=999)**
Mark only one oval.

- 1
- 2
- 999

68. **5.12. If a threshold was used, was it pre-specified? (1=yes, 2=no, unclear=999)**
Mark only one oval.

- 1
- 2
- 999

5. QUADAS Domain 2: Questions

Concerns regarding applicability

69. **5.13. Is there concern that the index test, its conduct, or interpretation differ from the review question? (3=low, 4=high, 999=unclear)**

Mark only one oval.

- 3
- 4
- 999

6. Domain 3: Reference standard

70. **6.1. What reference standard (s) was used to diagnose PTB? Check all that apply:**

Check all that apply.

- [ ] 1 Microbiologic tests only (in isolation; if used in combination answer in 17 b)
- [ ] 2 Composite reference standard(s) (two or three of the following standards): microbiologic tests, Clinically (eg. symptoms, non-response to anti-bacterials, response to anti-TB drugs) and X-ray
- [ ] 3 X-ray read by human readers only (If this is the answer, specify in Part 4)
- [ ] Other: ______________________

71. **6.1.1. if Composite reference standard(s) (two or three of the following standards): microbiologic tests, Clinically (eg. symptoms, non-response to anti-bacterials, response to anti-TB drugs) and X-ray, Indicate composite standards:**

____

____

____

____

____

72. **6.2 What microbiological test was used to diagnose PTB? Check all the apply**

Check all that apply.

- [ ] 1 Liquid culture (specify which kind question 6.2.2)
- [ ] 12 BACTEC 460
- [ ] 13 BacT/Alert 3D system
- [ ] 14 VersaTrek
- [ ] 15 Gottsacker
- [ ] 16 MGIT 960
- [ ] 2 Solid culture
- [ ] 21 Lowenstein-Jensen (L-J) media
- [ ] 22 MOD9
- [ ] 23 Middlebrook 7H10 and Middlebrook 7H117H10/7H11
- [ ] 3 NAAT
- [ ] 33 GeneXpert
- [ ] 4 LPA (a line probe assay to detect resistance to second-line anti-TB drugs)
- [ ] 5 Smear Ziehl Neelsen stain
- [ ] 6 Smear Flurochrome
- [ ] 7 Unknown/not reported
- [ ] Other: ____________________

73. **6.3. Were the microbiologic tests performed on all participants? (i.e. please ignore missing tests for this question, we are interested to know if the protocol called for the microbiologic tests on all participants) (1=yes, 2=no)**

Mark only one oval.

- ( ) 1
- ( ) 2

74. **6.3.1. If no (selection 2), microbiologic tests were only performed on a participants that met the following criteria:**

____
____
____
____
____

75. **6.4. Were the microbiologic tests performed regardless of the chest x-ray result? (i.e. please ignore missing tests for this question, we are interested to know if the protocol called for the microbiologic tests on all participants): (1=yes, 2=no)**

Mark only one oval.

- ( ) 1
- ( ) 2

76. **6.4.1 If no for the above question(selection 2), when was the microbiologic test performed?**
Mark only one oval.

- 1 The microbiologic tests were performed only if the CXR was abnormal as per CXR reader
- 2 The microbiologic tests were performed only if the CXR was normal as per CXR

77. **6.5. Were the microbiologic tests performed regardless of computer aided software result? (1=yes, 2=no)**
Mark only one oval.

- 1
- 2

78. **6.5.1. If no for the above question(selection 2), when was the microbiologic tests performed?**
Mark only one oval.

- 1 The microbiologic tests were performed only if the CAD was abnormal
- 2 The microbiologic tests were performed only if the CAD was normal

79. **6.6. How was a positive PTB defined in the study? (select all applicable):**
Check all that apply.

- [ ] 1 Sputum culture positive for M. tuberculosis
- [ ] 2 Sputum smear positive for acid-fast bacilli
- [ ] 3 Sputum GeneXpert MTB/RIF positive for M.
- [ ] 4 Tuberculosis complex
- [ ] 5 Sputum LPA positive for M. tuberculosis complex
- [ ] 6 Clinical criteria
- [ ] 7 Symptoms
- [ ] 8 Improvement with TB treatment
- [ ] 9 Chest x-rays read by human readers as being consistentwith TB (this means that chest x-rays read by humans was part of the reference standard)
- [ ] 10 Chest x-rays interpreted by human readers as showing improved with TB treatment (this means that chest x-rays read by humans was part of the reference standard)

Answer below questions only if the study compared software to humans:

80. **6.7. Were the human readers blinded to the diagnosis of the patient (i.e. Did the readers know whether the patient had TB or not) (1=yes, 2=no, 888=not reported)?**
Mark only one oval.

- 1
- 2
- 888

81. **6.8. Were human readers blinded to software results prior to reading the conventional radiograph? (i.e. Did the readers know the software result before reading the x-ray?) (1=yes, 2=no)**

Mark only one oval.

- 1
- 2

6. QUADAS Domain 3: Questions

Risk of bias

82. **6.9. Briefly describe the reference standard and how it was conducted and interpreted:**

83. **6.10. Is the reference standard likely to correctly classify the target condition? (1=yes, no=2, 999=unclear)**

Mark only one oval.

- 1
- 2
- 999

84. **6.11. Were the reference standard results interpreted without knowledge of the results of the index test? (1=yes, no=2, 999=unclear)**

Mark only one oval.

- 1
- 2
- 999

85. **6.12. Could the reference standard, its conduct, or its interpretation have introduced bias? (risk: 3=low, 4=high, 999=unclear)**

Mark only one oval.

- 3
- 4
- 999

6. QUADAS Domain 3: Questions

Concerns regarding applicability

86. **6.13. Is there concern that the target condition as defined by the reference standard does not match the review question? (concern: 3=low, 4=high, 999=unclear)**

Mark only one oval.

- 3
- 4
- 999

7. PROFILES of HUMAN X-RAY READERS (SKIP IF X-RAYS NOT READ BY HUMANS)

87. **7.1.Number of readers involved:**

88. **7.2. Were the readers trained specifically for the study? (1=yes, no=2)**
Mark only one oval.

- 1
- 2

89. **7.2.1. If 1, how were they trained?**

90. **7.3. Was one of the readers used as a reference ("expert") against which the other readers were compared? (1=yes, no=2)**
Mark only one oval.

- 1
- 2

91. **7.3.1. if 1, Years of experience,Title, and Educational qualification**

7.4.Characteristics of non- expert readers (if applicable)

92. **7.4.1. Enter the number of non-expert readers**

93. **7.4.2. Average years of experience**

94. **7.4.3. Title**

95. **7.4.4. Educational qualification:**

96. **7.4.5. Number with previous experience in reading chest x-rays**

8. Domain 4: Flow and Timing

97. **8.1. Was there an interval of more than 7 days between the performance of the chest x-ray and the microbiologic reference tests? (1=yes, 2=no, 888=not reported)**
Mark only one oval.

- 1
- 2
- 888

98. **8.1.1. If 1, describe reason**

8. QUADAS Domain 4: Questions

Risk of Bias

99. **8.2. Briefly describe any patients who did not receive the index test(s) and/or reference standard or who were excluded from the 2x2 table (refer to flow diagram).**

100. **8.3. Briefly describe the time interval and any interventions between index test(s) and reference standard:**

101. **8.4. Was there an appropriate interval between index test(s) and reference standard? (1=yes, 2=no, 999=unclear)**
Mark only one oval.

- 1
- 2
- 999

102. **8.5. Did all patients receive a reference standard? (1=yes, 2=no, 999=unclear)**
Mark only one oval.

- 1
- 2
- 999

103. **8.6. Did patients receive the same reference standard? (1=yes, 2=no, 999=unclear)**
Mark only one oval.

- 1
- 2
- 999

104. **8.7. Were all patients included in the analysis? (1=yes, 2=no, 999=unclear)**
Mark only one oval.

- 1
- 2
- 999

105. **8.8. Could the patient flow have introduced bias? (risk: 3=low, 4=high, 999=unclear)**
Mark only one oval.

- 3
- 4
- 999

9. Sensitivity and Specificity

106. **9.1. Is the total number of participants reported in the results analysis EQUAL to the number of participants enrolled? (1=yes, 2=no)**
Mark only one oval.

- 1
- 2

107. **9.1.1. If 2, Number of enrolled participants, Number of participants in results analysis , and please explain why**

108. **9.2. Was interpretation categorical or continuous or both?**
Mark only one oval.

- 1 Categorical
- 2 Continuous
- 3 Both

109. **9.2.1. If categorical:**
Mark only one oval.

- 1 normal vs. abnormal
- 2 PTB suspected vs. no PTB suspected
- Other: ____________________

110. **9.2.2. If continuous what was the scale used?**

____

111. **9.3. Were the criteria to distinguish between positive (abnormal) and negative (normal) chest x-rays pre- specified? (1=yes, 2=no)**
Mark only one oval.

- 1
- 2

112. **9.3.1. If 1, Specify**

____

113. **9.4. What was the reported specificity? (e.g. 0.97)**

____

114. **9.4.1. Lower bound 95% CI**

____

115. **9.4.2. Upper bound 95%CI**

____

116. **9.5. What was the reported sensitivity?(e.g. 0.97)**

____

117. **9.5.1.lower bound 95% CI**

____

118. **9.5.2. upper bound 95%CI**

____

119. **9.6. What was the reference standard used? (Check all the apply)**
Check all that apply.

- [ ] 1 Microbiologic - Culture
- [ ] 2 Microbiologic - PCR
- [ ] 3 Microbiologic - AFB smear
- [ ] 4 Radiologic
- [ ] 5 Clinical (diagnosis of PTB)

120. **9.7. What was the AUC if reported?**

10. TRUE POSITIVES AND NEGATIVES (microbiological reference standard)

10.1. Reference standard used

121. **10.1.1. Number of CAD Positive and microbiological reference Standard-Positive**

122. **10.1.2. Number of CAD Positive and microbiological reference Standard-Negative**

123. **10.1.3. Number of CAD Negative and microbiological reference Standard-Positive**

124. **10.1.4. Number of CAD Negative and microbiological reference Standard-Negative**

10.2. Children (age < 15 years)

125. **10.2.1. Number of CAD Positive and microbiological reference Standard-Positive**

126. **10.2.2. Number of CAD Positive and microbiological reference Standard-Negative**

127. **10.2.3. Number of CAD Negative and microbiological reference Standard-Positive**

128. **10.2.4. Number of CAD- Negative and microbiological reference Standard-Negative**

10.3. Adults (age > 15 years)

129. **10.3.1. Number of CAD Positive and microbiological reference Standard-Positive**

130. **10.3.2. Number of CAD Positive and microbiological reference Standard-Negative**

131. **10.3.3. Number of CAD Negative and microbiological reference Standard-Positive**

132. **10.3.4. Number of CAD Negative and microbiological reference Standard-Negative**

10.4. PLWH only

133. **10.4.1. Number of CAD Positive and microbiological reference Standard-Positive**

134. **10.4.2. Number of CAD Positive and microbiological reference Standard-Negative**

135. **10.4.3. Number of CAD Negative and microbiological reference Standard-Positive**

136. **10.4.4. Number of CAD Negative and microbiological reference Standard-Negative**

10.5. Uninfected HIV participants

137. **10.5.1. Number of CAD Positive and microbiological reference Standard-Positive**

138. **10.5.2. Number of CAD Positive and microbiological reference Standard-Negative**

139. **10.5.3. Number of CAD Negative and microbiological reference Standard-Positive**

140. **10.5.4. Number of CAD Negative and microbiological reference Standard-Negative**

11. TRUE POSITIVES AND NEGATIVES (2 Composite reference standard(s)

Two or three of the following standards: microbiologic tests, Clinically (eg. symptoms, non-response to anti-bacterials, response to anti-TB drugs), and X-ray

11.1. Reference standard used

141. **11.1.1. Number of CAD Positive and composite reference Standard-Positive**

142. **11.1.2. Number of CAD Positive and composite reference Standard-Negative**

143. **11.1.3. Number of CAD Negative and composite reference Standard-Positive**

144. **11.1.4. Number of CAD Negative and composite reference Standard-Negative**

11.2. Children (age < 15 years)

145. **11.2.1. Number of CAD Positive and composite reference Standard-Positive**

146. **11.2.2. Number of CAD Positive and composite reference Standard-Negative**

147. **11.2.3. Number of CAD Negative and composite reference Standard-Positive**

148. **11.2.4. Number of CAD4TB- Negative and composite reference Standard-Negative**

11.3. Adults (age > 15 years)

149. **11.3.1. Number of CAD Positive and composite reference Standard-Positive**

150. **11.3.2. Number of CAD Positive and composite reference Standard-Negative**

151. **11.3.3. Number of CAD Negative and composite reference Standard-Positive**

152. **11.3.4. Number of CAD Negative and composite reference Standard-Negative**

11.4. PLWH only

153. **11.4.1. Number of CAD Positive and composite reference Standard-Positive**

154. **11.4.2. Number of CAD Positive and composite reference Standard-Negative**

155. **11.4.3. Number of CAD Negative and composite reference Standard-Positive**

156. **11.4.4. Number of CAD Negative and composite reference Standard-Negative**

11.5. Uninfected HIV participants

157. **11.5.1. Number of CAD Positive and composite reference Standard-Positive**

158. **11.5.2. Number of CAD Positive and composite reference Standard-Negative**

159. **11.5.3. Number of CAD Negative and composite reference Standard-Positive**

160. **11.5.4. Number of CAD Negative and composite reference Standard-Negative**

12. TRUE POSITIVES AND NEGATIVES: X-ray read by human readers only

Reference standard: human readers

12.1. Reference standard used

161. **12.1.1. Number of CAD Positive and human reader reference Standard-Positive**

162. **12.1.2. Number of CAD Positive and human reader reference Standard-Negative**

163. **12.1.3. Number of CAD Negative and human reader reference Standard-Positive**

164. **12.1.4. Number of CAD Negative and human reader reference Standard-Negative**

12.2. Children (age < 15 years)

165. **12.2.1. Number of CAD Positive and human reader reference Standard-Positive**

166. **12.2.2. Number of CAD Positive and human reader reference Standard-Negative**

167. **12.2.3. Number of CAD Negative and human reader reference Standard-Positive**

168. **12.2.4. Number of CAD- Negative and human reader reference Standard-Negative**

12.3. Adults (age > 15 years)

169. **12.3.1. Number of CAD Positive and human reader reference Standard-Positive**

170. **12.3.2. Number of CAD Positive and human reader reference Standard-Negative**

171. **12.3.3. Number of CAD Negative and human reader reference Standard-Positive**

172. **12.3.4. Number of CAD Negative and human reader reference Standard-Negative**

12.4. PLWH only

173. **12.4.1. Number of CAD Positive and human reader reference Standard-Positive**

174. **12.4.2. Number of CAD Positive and human reader reference Standard-Negative**

175. **12.4.3. Number of CAD Negative and human reader reference Standard-Positive**

176. **12.4.4. Number of CAD Negative and human reader reference Standard-Negative**

12.5. Uninfected HIV participants

177. **12.5.1. Number of CAD Positive and human reader reference Standard-Positive**

178. **12.5.2. Number of CAD Positive and human reader reference Standard-Negative**

179. **12.5.3. Number of CAD Negative and human reader reference Standard-Positive**

180. **12.5.4. Number of CAD Negative and human reader reference Standard-Negative**

13.CONCLUSION

181. **13. What was the conclusion from the study?**
