# Supplementary material for: A systematic review of the diagnostic accuracy of artificial intelligence-based computer programs to analyze chest x-rays for pulmonary tuberculosis
Source: PLoS One. 2019 Sep 3;14(9):e0221339. doi: 10.1371/journal.pone.0221339 (PMC6719854; doi:10.1371/journal.pone.0221339)
Supplement: S1 Table — CAD, computer aided diagnosis; yrs, years; NR, not reported; TB, tuberculosis; HIV, human immunodeficiency virus *This is the median, the mean age was not reported. (PDF) [file pone.0221339.s004.pdf]

S1 Table. Demographics of CAD4TB studies with microbiologic reference standard

Reference	All	Gender		Age			Previous TB		HIV		TB prev-alence N (%)
		Male (%)	Female (%)	< 15 yrs(%)	≥ 15 yrs(%)	average age	Yes N (%)	No N (%)	Positive N (%)	Negative N (%)	
**CAD4TB Triage**											
Zaidi et al, 2018	6090	3018 (50)	3072 (50)	0 (0)	6090 (100)	38.9*	NR	NR	NR	NR	925 (15)
Rahman et al, 2017	17066	11368 (67)	5698 (33)	0 (0)	17066 (100)	NR	NR	NR	NR	NR	2623 (15)
Melendez et al, 2016	392	240 (61)	152 (39)	0 (0)	392 (100)	40	NR	NR	130 (33)	262 (67)	73 (19)
Breuninger et al, 2014	861	433 (50)	428 (50)	0 (0)	861 (100)	42	144 (17)	717 (83)	379 (44)	482 (46)	194 (23)
Muyoyeta et al, 2014	350	215 (61)	135 (38)	NR	NR	36.5	78 (22)	272 (78)	190 (54)	166 (57)	96 (33)
Maduskar et al, 2013	161	119 (74)	42 (26)	0 (0)	161 (100)	35.8	NR	NR	110 (68)	51 (32)	97 (60)
**CAD4TB Screening**											
Koesoemadinata et al, 2018	346	151 (44)	195 (56)	0 (0)	346 (100)	59.3	38 (11)	308 (89)	1 (0.3)	3 (0.8)	9 (3)
Melendez et al, 2018	38961	NR	NR	NR	NR	NR	NR	NR	NR	NR	87 (0.2)
Melendez et al, 2017	23838	10440 (44)	13398 (56)	0 (0)	23838	36	NR	NR	NR	NR	106 (0.4)
Muyoyeta et al, 2017	919	370 (40)	549 (60)	NR	NR	15	57 (6)	862 (94)	138 (15)	781 (85)	32 (7)

CAD, computer aided diagnosis; yrs, years; NR, not reported; TB, tuberculosis; HIV, human immunodeficiency virus

*This is the median, the mean age was not reported.
