# Supplementary material for: A systematic review of the diagnostic accuracy of artificial intelligence-based computer programs to analyze chest x-rays for pulmonary tuberculosis
Source: PLoS One. 2019 Sep 3;14(9):e0221339. doi: 10.1371/journal.pone.0221339 (PMC6719854; doi:10.1371/journal.pone.0221339)
Supplement: S2 Table — NR, not reported; CAD, computer aided diagnosis; NAAT, nucleic acid amplification test * Patients with an abnormal CXR as per radiologist reading, or presumptive TB based on TB symptoms received culture ** Patients with a normal CXR by CAD received an AFB smear, while patients with an abnormal CXR as per CAD received NAAT. (PDF) [file pone.0221339.s005.pdf]

S2 Table. Selection, enrolment of CAD4TB studies with microbiologic reference standard

Reference	Eligible, N	Enrolled, N (% of eligible)	Reported for assessment of CAD4TB, N (% of enrolled)	Smear positive, N (% of reported)	Culture or NAAT positive N (% of reported)
**CAD4TB Triage**					
Zaidi et al, 2018	NR	6845	6090 (89.0)	NR	925 (15.2)
Rahman et al, 2017	18036	17134 (95)	17066 (99)	NR	2623 (15)
Melendez et al, 2016	NR	392	392 (100)	NR	73 (19)
Philipsen et al, 2015	758	419 (55)	388 (93)	NR	133 (34)
Muyoyeta et al, 2015	10618	9509 (90)	9482 (99)	8 (<1) *	2090 (22) *
Breuninger et al, 2014	894	861 (96)	566 (66)	146 (17)	194 (23)
Muyoyeta et al, 2014	458	391 (85)	350 (90)	52 (13)	96 (35)
Maduskar et al, 2013	NR	161	161 (100)	69 (43)	97 (60)
**CAD4TB Screening**					
Koesoemadinata et al, 2018	866	794	346 (44)	NR	12 (1.56)
Melendez et al, 2018	NR	39328	38961 (99)	NR	87 (0.2)
Melendez et al, 2017	46099	25805 (56)	23838 (92)	NR	106 (0.4)*
Muyoyeta et al, 2017	919	865 (94)	865 (100)	0*	19 (2)**
Steiner et al, 2015	516	511 (99)	511 (100)	NR	NR

NR, not reported; CAD, computer aided diagnosis; NAAT, nucleic acid amplification test
* Patients with an abnormal CXR as per radiologist reading, or presumptive TB based on TB symptoms received culture
** Patients with a normal CXR by CAD received an AFB smear, while patients with an abnormal CXR as per CAD received NAAT
