# Supplementary material for: A systematic review of the diagnostic accuracy of artificial intelligence-based computer programs to analyze chest x-rays for pulmonary tuberculosis
Source: PLoS One. 2019 Sep 3;14(9):e0221339. doi: 10.1371/journal.pone.0221339 (PMC6719854; doi:10.1371/journal.pone.0221339)
Supplement: S3 Table — AMPATH, Academic Model Providing Access to Healthcare; CH, Shenzhen Hospital, China; F&T, Find and Treat; IN, Indian collection New Delhi; JSRT, Japanese Society of Radiology; KIT, Korean Institute of Tuberculosis; MC, Montgomery County; YU AWHE, Yonsei University Annual Worker's health examination; SNUH, Seoul National University Hospital; TJH, Thomas Jefferson Hospital dataset; U, unclear; H, high; NA, not applicable; L, low * Calgary dataset included preselected “typical PTB” images ** JSRT data set does not include PTB cases, but rather comprises images with single pulmonary nodules, confirmed by computed tomography and histology as either benign or pathologic. (PDF) [file pone.0221339.s006.pdf]

S3 Table. Quality assessment of Datasets used to test and train CAD software of Development Studies: risk of bias and applicability concerns

Reference	Risk of Bias		Applicability
	Patient Selection	Reference Test	Reference Test
AMPATH	U	U	U
Calgary dataset*	L	H	H
CH	H	H	H
F&T	L	L	L
Gambian dataset	L	H	H
IN	U	H	H
JSRT**	NA	NA	NA
Kenyan dataset	U	H	H
KIT dataset	U	U	U
Large Zambian dataset	L	H	H
MC	U	H	H
SNUH	H	L	L
Sub-Saharan Africa	L	H	H
TB-NEAT	L	L	L
TJH	U	L	L
Tanzania dataset	L	H	H
YU AWHE	L	U	U
Zambian dataset	L	H	H

AMPATH, Academic Model Providing Access to Healthcare; CH, Shenzhen Hospital, China; F&T, Find and Treat; IN, Indian collection New Delhi; JSRT, Japanese Society of Radiology; KIT, Korean Institute of Tuberculosis; MC, Montgomery County; YU AWHE, Yonsei University Annual Worker's health examination; SNUH, Seoul National University Hospital; TJH, Thomas Jefferson Hospital dataset; U, unclear; H, high; NA, not applicable; L, low

* Calgary dataset included preselected “typical PTB” images

** JSRT data set does not include PTB cases, but rather comprises images with single pulmonary nodules, confirmed by computed tomography and histology as either benign or pathologic
