# Supplementary material for: A systematic review of the diagnostic accuracy of artificial intelligence-based computer programs to analyze chest x-rays for pulmonary tuberculosis
Source: PLoS One. 2019 Sep 3;14(9):e0221339. doi: 10.1371/journal.pone.0221339 (PMC6719854; doi:10.1371/journal.pone.0221339)
Supplement: S4 Table — (PDF) [file pone.0221339.s007.pdf]

S4 Table. Quality assessment (QUADAS 2) summary of development studies: risk of bias & applicability concerns.

	Risk of Bias				Applicability Concerns		
	Patient Selection	Index Test	Reference Standard	Flow and Timing	Patient Selection	Index Test	Reference Standard
Alfadhli,	Unclear	High	High	Unclear	Unclear	High	High
Arzhaeva,	Low	High	High	Unclear	Low	High	High
Chauhan,	Unclear	High	High	Unclear	Unclear	High	High
Ding,	Unclear	High	High	Unclear	Unclear	High	High
Fatima,	Unclear	High	High	Low	Unclear	High	High
Gabriella,	High	High	High	Unclear	Unclear	High	High
Heo,	Low	High	Low	Low	Low	High	Low
Hogeweg,	Low	High	Low	Low	High	High	Low
Hogeweg,a	High	High	High	Unclear	High	High	High
Hogeweg,b	Unclear	High	High	Unclear	Unclear	High	High
Hwang,	Unclear	High	High	High	Unclear	High	High
Hwang,a	High	High	Low	Low	Unclear	High	Low
Jaeger	High	High	High	Low	High	High	High
Jaeger 2012	High	High	High	Low	High	High	High
Karargyris,	High	High	High	Low	High	High	High
Lakhani,	Unclear	High	High	Unclear	Unclear	High	High
Lopes,	Unclear	High	High	Low	Unclear	High	High
Maduskar,	Low	High	High	Low	Low	High	High
Melendez,	Low	High	High	Low	Low	High	High
Melendez,a	Low	High	High	Low	Low	High	High
Melendez,b	Low	High	High	Low	Low	High	High
Mouton,	Low	High	High	Low	Low	High	High
Noor,	Unclear	High	Unclear	Unclear	High	High	Unclear
Poornimadevi,	High	High	High	Unclear	High	Unclear	High
Rajaraman, NLM	High	Low	High	Unclear	High	Low	High
Santosh,	Unclear	High	High	Low	High	High	High
Santosh,a	High	High	High	Unclear	Unclear	High	High
Shen,	High	High	High	Low	High	High	High
Sivaramakrishnan,	High	High	High	Unclear	Unclear	High	High
Sundaram,	Unclear	High	High	Unclear	Unclear	High	High
Udayakumar,	Unclear	High	High	Unclear	Unclear	High	High
Vajda,	High	High	High	Unclear	High	High	High
Xu,	High	High	High	Unclear	High	High	High

High Unclear Low
