# Supplementary material for: A systematic review of the diagnostic accuracy of artificial intelligence-based computer programs to analyze chest x-rays for pulmonary tuberculosis
Source: PLoS One. 2019 Sep 3;14(9):e0221339. doi: 10.1371/journal.pone.0221339 (PMC6719854; doi:10.1371/journal.pone.0221339)
Supplement: S5 Table — (PDF) [file pone.0221339.s008.pdf]

S5 Table. Quality assessment (QUADAS 2) summary of clinical studies: risk of bias and applicability concerns.

	Risk of Bias				Applicability Concerns		
	Patient Selection	Index Test	Reference Standard	Flow and Timing	Patient Selection	Index Test	Reference Standard
Breuninger 2014	−	−	+	+	−	−	+
Koesoemadinata, 2018	+	−	+	+	+	−	+
Maduskar 2013	−	−	+	?	−	−	+
Melendez, 2018	+	?	+	+	+	+	+
Melendez 2016	+	+	+	?	+	+	+
Melendez 2017	+	−	+	+	+	−	+
Muyoyeta 2014	+	+	+	+	+	+	+
Muyoyeta 2015	+	+	−	−	+	+	−
Muyoyeta 2017	+	+	−	−	+	+	−
Philipsen 2015	−	−	+	?	?	−	+
Rahman 2017	+	−	+	+	+	−	+
Steiner 2015	−	−	−	+	−	−	−
Zaidi, 2018	+	−	+	+	+	−	+

− High ? Unclear + Low
